# Comparing lumbo-pelvic kinematics in people with and without back pain: a systematic review and meta-analysis

**DOI:** 10.1186/1471-2474-15-229

**Published:** 2014-07-10

**Authors:** Robert A Laird, Jayce Gilbert, Peter Kent, Jennifer L Keating

**Affiliations:** 1 Department of Physiotherapy, Monash University, PO Box 527, Frankston, VIC 3199, Australia; 2 Peak MSK Physiotherapy, Suite 4/544 Hampton St, Hampton, VIC 3188, Australia; 3 Department of Sports Science and Clinical Biomechanics, University of Southern Denmark, Odense 5230, Denmark; 4 Research Department, Spine Centre of Southern Denmark, Lillebaelt Hospital, Institute of Regional Health Services Research, University of Southern Denmark, Middelfart 5500, Denmark; 5 7 Kerry Rd, Warranwood, Melbourne, VIC 3134, Australia

**Keywords:** Low back pain, Movement disorders, Posture, Range of movement, Lordosis, Proprioception

## Abstract

**Background:**

Clinicians commonly examine posture and movement in people with the belief that correcting dysfunctional movement may reduce pain. If dysfunctional movement is to be accurately identified, clinicians should know what constitutes normal movement and how this differs in people with low back pain (LBP). This systematic review examined studies that compared biomechanical aspects of lumbo-pelvic movement in people with and without LBP.

**Methods:**

MEDLINE, Cochrane Central, EMBASE, AMI, CINAHL, Scopus, AMED, ISI Web of Science were searched from inception until January 2014 for relevant studies. Studies had to compare adults with and without LBP using skin surface measurement techniques to measure lumbo-pelvic posture or movement. Two reviewers independently applied inclusion and exclusion criteria, and identified and extracted data. Standardised mean differences and 95% confidence intervals were estimated for group differences between people with and without LBP, and where possible, meta-analyses were performed. Within-group variability in all measurements was also compared.

**Results:**

The search identified 43 eligible studies. Compared to people without LBP, on average, people with LBP display: (i) no difference in lordosis angle (8 studies), (ii) reduced lumbar ROM (19 studies), (iii) no difference in lumbar relative to hip contribution to end-range flexion (4 studies), (iv) no difference in standing pelvic tilt angle (3 studies), (v) slower movement (8 studies), and (vi) reduced proprioception (17 studies). Movement variability appeared greater for people with LBP for flexion, lateral flexion and rotation ROM, and movement speed, but not for other movement characteristics. Considerable heterogeneity exists between studies, including a lack of detail or standardization between studies on the criteria used to define participants as people with LBP (cases) or without LBP (controls).

**Conclusions:**

On average, people with LBP have reduced lumbar ROM and proprioception, and move more slowly compared to people without LBP. Whether these deficits exist prior to LBP onset is unknown.

## Background

Observation of lumbo-pelvic movement and posture is a basic component of the physical examination of people with low back pain (LBP) [1-4] partly due to a common belief held by clinicians that identifying and correcting movement/postural aberration can improve pain and activity limitation [2,5,6]. Examination of lumbo-pelvic movement typically includes basic kinematic assessments, such as range of movement (ROM) and posture. It may also include higher order kinematics such as temporal and sequential patterns during physiological movements, proprioception, muscle activation patterns, postural sway and/or complex functional movements such as walking or lifting. If clinicians aim to ‘normalise’ dysfunctional movement, they need an empirical basis for (i) differentiating between normal and dysfunctional movement, and (ii) determining whether correction of dysfunctional movement might reduce pain and activity limitation. Measurement of movement and posture has been problematic in typical clinical settings due to limitations (practicality, accuracy, comprehensiveness, reliability) of simple measurement tools such as goniometers, tape measures and inclinometers [7]. Advances in technology are creating new opportunities, available for use in typical clinical settings, that measure comprehensive information about the relationship between movement/posture and pain [8-10].

Measurements reported in studies of lumbo-pelvic kinematics, such as ROM, vary considerably. This variability may be due to differences in measurement instruments or methods [11], biological differences in true range of movements, or errors in measurements. Intolo [12], in a systematic review into the effect of age on ROM, performed a meta-analysis of mean scores for lumbar ROM for 20-29 year olds. Across studies, the lowest reported group mean score for flexion was 24 ± 7° [13] while the highest was 75 ± 10° [14]. Similarly, mean scores for extension ranged from 13 ± 8° [13] to 41 ± 10° [15]. These large differences between studies are unlikely to be due to biological differences alone. Milosavljevic et al. [13] provided ROM estimates using a photographic method, Russell et al. [14] used an Isotrak system and Fitzgerald et al. [15] used a tape-measure (Schober) method [16]; such method differences are likely to account for a large proportion of observed differences. Similar variation is seen for axial rotation and lateral flexion movements. Extreme variations in reported ROM measurements limit confidence in clinical interpretations or treatment decisions based on measurements of an individual.

A search for reviews on what is known about typical movement in people with and without LBP identified one review on postural sway [17], and one review on age-related changes to lumbar spine ROM [12]. The qualitative review on postural sway, reported that 14 of 16 included papers concluded that people with LBP have greater postural sway excursion when compared to people without LBP. The review on age-related change to lumbar ROM reported a reduction in ROM associated with increasing age but did not include people with LBP and did not report mean ROM data. No reviews were found comparing people with and without LBP on any other movement characteristics. Therefore, we designed this review to systematically investigate and compare typical lumbo-pelvic movement differences between people with and without LBP, focusing on ROM, movement sequence and speed, a movement related measure of proprioception (positioning/re-positioning accuracy), pelvic tilt angles (in standing and sitting), and segmental body contributions to movement (lumbar versus hip contributions). We also compared differences in variability between the two groups.

## Methods

### Study selection: inclusion and exclusion criteria

For inclusion in the review, studies had to (i) assess adults >17 years; (ii) use non-invasive measurement systems (i.e. did not use measurements such as X-rays, CT scans); (iii) apply the same procedures to measure people with low back +/-leg pain (LBP group) and people without LBP (NoLBP group), (iv) measure at least one of lumbar lordosis, lumbar range of motion (ROM), speed/acceleration/timing of lumbar +/- hip movement, pelvic tilt angle (as measured by a line drawn from anterior to posterior superior iliac spines with an angle formed relative to horizontal, measured in sitting or standing), pelvic tilt ROM (defined as a range from maximum anterior tilt to maximum posterior tilt), usual sitting pelvic tilt position (i.e. relative to full anterior tilt), lumbar compared with hip contributions to ROM, lumbo-pelvic proprioceptive position/re-position accuracy; (v) report appropriate measurement means (or other point estimates) and variance estimates or data that enable estimation of these values. In order to fully survey published research on lumbo-pelvic movement, no specific definitions of back pain or control (NoLBP) groups were required but the definitions of LBP group, pain intensity and NoLBP group within each study were extracted. Studies were excluded if they (i) included people who had lumbar surgery in the previous 12 months; (ii) reported that subjects had fracture, neurological conditions, metabolic disease, neoplasm, or scoliosis; (iii) measured only whole body movement such as distance from finger-tip-to-floor or (iv) reported insufficient data, e.g. did not report measures of variability. Lead authors were contacted to obtain additional data as required.

### Data sources

**Figure 1:**
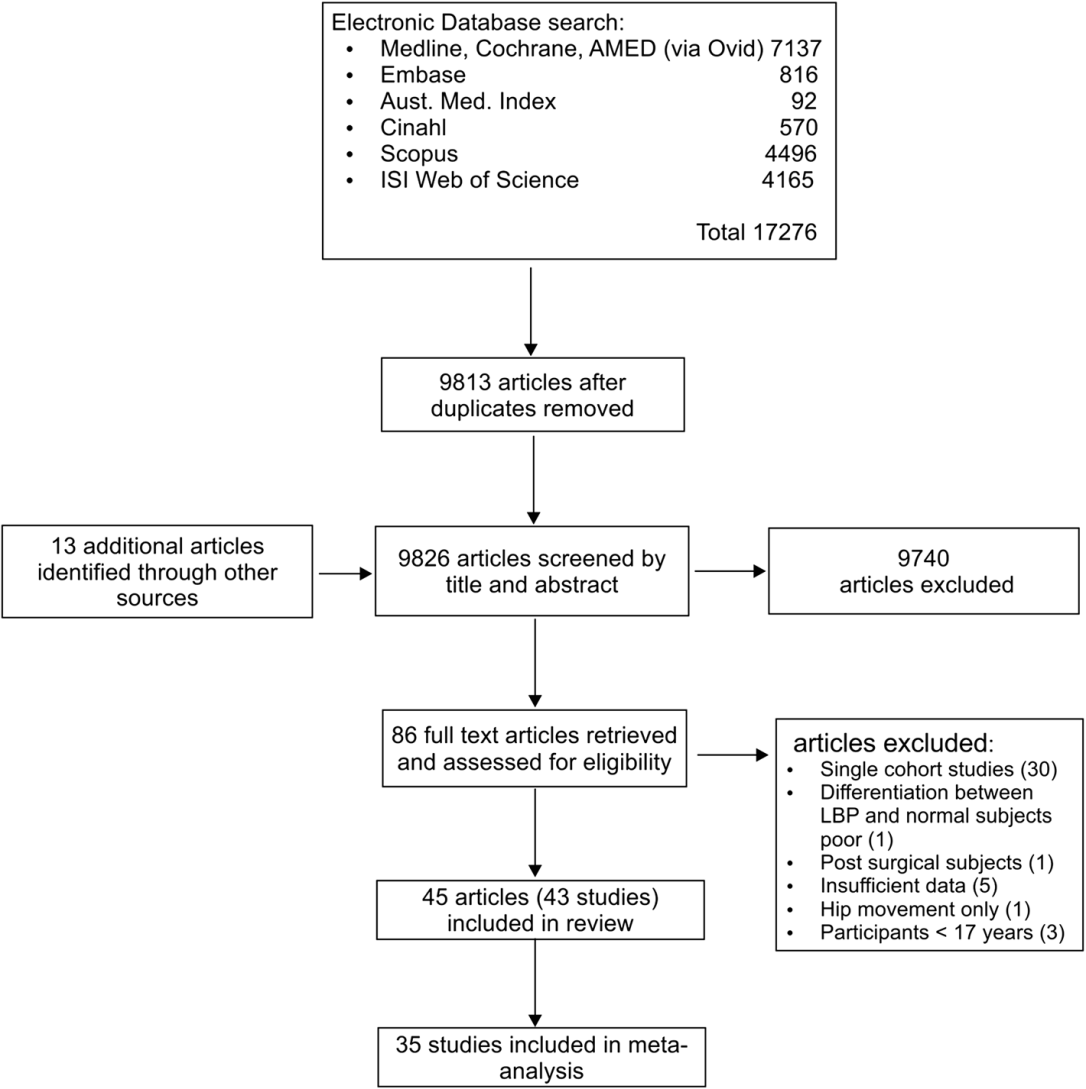
Flow diagram of study inclusion.

Eight electronic databases (MEDLINE, Cochrane Central Register of Controlled Trials (Central), EMBASE, AMI, CINAHL, Scopus, AMED, ISI Web of Science) were searched from inception until January 2014 using a broad search strategy based on relevant medical subject heading (MeSH) terms [18] (see Additional file 1). The search yield was initially screened for eligibility by one reviewer (RL) on title and abstract to remove duplicates and clearly unrelated articles. Following this, two reviewers (RL and JG) independently identified potentially relevant articles based on title and abstract. Full text articles were retrieved and checked for compliance with inclusion and exclusion criteria. References of potentially relevant reports were reviewed for additional papers. Consensus by discussion was then reached on article inclusion. Where disagreement occurred, a third reviewer (JK) was included and discussion continued until consensus was achieved. A flow diagram of the study selection process based on PRISMA recommendations [19] is seen in Figure 1.

### Data extraction and study quality assessment

A checklist for data extraction was developed based on those used in a similar review [12] and published quality assessment tools [20-22]. The following study details were extracted: participant age, sex, and source characteristics, inclusion/exclusion criteria, training of testers (profession, experience), measurement methods and procedures (instrument used, instructions to participants, position of testing), the movement characteristics assessed (e.g. range, speed, relative contributions of body segments), pain/function measures, measurements for those with and without back pain (e.g. means, standard deviations). A quality assessment tool, using a similar approach to Mieritz [23], was constructed to determine how each study accounted for possible sources of bias, and if the study provided details on: (i) study population (age, sex, BMI, source), (ii) participant LBP (chronicity, +/- leg pain, specific versus non-specific, pain intensity and activity limitation scores), (iii) measurement procedures (i.e. detail that would enable accurate replication of the experiment, instrument description, standardised movement instructions, movement process description e.g. fixed or free pelvis), (iv) blinding of assessors to the presence of back pain (yes/no), and (vi) whether the same assessment procedures were applied to participants with and without back pain (see Additional file 2). Two reviewers independently extracted data, compared results and resolved differences through discussion.

### Data synthesis and analysis

Study details were extracted and summarised (Additional files 3 and 4). For each comparison, standardised mean differences (SMD) between groups with and without LBP were calculated using Revman software [24]. Pooled estimates of overall differences were calculated by meta-analysis of studies that measured a kinematic characteristic using comparable methods. For example studies on flexion ROM were included in a meta-analysis if subjects were standing using angular measurement but excluded if subjects were in other positions (i.e. four point kneeling) or if linear/distance measurements were used. Reasons for exclusion from meta-analysis are found in Additional file 3. A random effects model was used for pooling where fixed effects modeling indicated statistical heterogeneity of the data (Mantel-Haenszel method), as determined by chi-squared and I^2^ statistics; otherwise the results of fixed effects modeling was reported [25,26].

We also planned to explore the within-group variability in each measured movement characteristic. To estimate whether variability for each movement characteristic differed between groups with and without LBP, a coefficient of variation (CoV) [27] (standard deviation in measurements divided by the group mean) was calculated for each movement parameter using those studies included in the relevant meta-analysis. CoVs were averaged after weighting for sample size. Differences between groups were examined by creating a ratio of weighted averages where ratios >1 indicate greater variability for those with LBP and ratios <1 indicate greater variability for those without LBP. Significant differences in pooled CoVs were examined by estimating 95% confidence intervals for observed ratios. The correlation (Pearson’s r) between effect size and study quality was calculated using STATA (version 12, Stata Corp, College Station, Texas USA).

## Results

### Search yield

The search identified 17,276 potentially relevant articles with 13 articles identified from bibliographies of related articles or other sources. Following screening of title and abstract, full texts of 86 articles were retrieved. Forty three studies (45 articles) met the inclusion criteria [28-70]. The study selection process is shown in Figure 1. A summary of included studies can be seen in Additional file 3. A list of studies retrieved in full text and subsequently excluded, and reasons for exclusion, are available from the first author on request.

#### Types of studies found

Included studies were grouped in categories: lordosis [31,32,38,47-49,57,58], range of movement (ROM) [29,30,34,37-42,44,47,50-54,56-59,69,71], relative hip and lumbar contribution to trunk flexion/extension [34,40,50,52,61,71], pelvic angle/relative position and ROM [31,32,57,58], speed/acceleration of lumbar movement [28,34,37,39,41,42,50,71], and proprioception (repositioning accuracy) [33,35,45,46,53,55,60-68,70,72]. Additional file 4 summarises the characteristics of included studies.

#### Definition of LBP and NoLBP groups

##### Case definition (LBP)

Of the 43 studies included, 48% provide no detail on diagnostic criteria, 37% defined their LBP participants as non-specific, and the remaining 15% used either a Quebec [73] or a movement based classification (see Additional file 5 for details). Fifty-six percent reported pain intensity scores.

##### Control definition (NoLBP)

A definition of control participants was provided by 60% of the 43 studies. Those definitions were highly variable, ranging from vague descriptions such as ‘no current pain’ (16%), six-months (14%), 12-months (14%) or 24-months (7%) pain free to ‘no LBP ever’ (9%).

#### Quality assessment

Table 1 lists the domains identified as potential sources of bias in the included studies and the percentage compliance with each item. No studies attempted blinding of assessors to group status, and only one study reported standardizing instructions to participants. The potential influence of study quality on reported differences between groups was examined for all groups. There was no significant correlation observed between total quality assessment scores and the magnitude of SMDs in measurements for those with and without LBP (r = 0.03), There was also no significant difference between individual items of quality assessment and the size of SMD. Results for individual studies are available in Additional file 5.

**Table 1:** **Quality assessment summary (see Additional files **2** and **5** for item decision rules and scores for each included study)**

	**Quality assessment domains**	**Percentage of studies scoring yes**
	**Selection bias**	
1.	Was the study population adequately described?	57%
2.	Where both groups drawn from the same population?	39%
3.	Were both groups comparable for age, sex, BMI/weight	54%
4.	Was pain intensity and/or activity limitation described for LBP group?	56%
5.	Was an attempt made to define back pain characteristics?	34%
	**Measurement and outcome bias**	
6.	Did the method description enable accurate replication of the measurement procedures	90%
7.	Was the measurement instrument adequately described?	95%
8.	Was a system for standardising movement instructions reported?	37%
9.	Were assessors trained in standardised measurement procedure?	2%
10.	Did the same assessors test those with and without back pain	17%
11.	Were assessors blinded as to which group subjects were in?	0%
12.	Was the same assessment procedure applied to those with and without back pain?	93%
	**Data presentation**	
13.	Were between-group statistical comparisons reported for at least one key outcome	94%

### Movement characteristics

#### Lordosis

**Figure 2:**
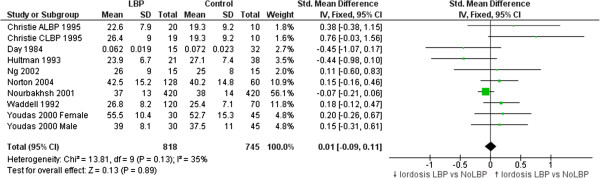
**Studies comparing lordosis in LBP versus NoLBP groups.** Means & standard deviations (SD) are in degrees with the exception of Day et al. [32] who used an algebraic computation based on linear measurement.

A meta-analysis of eight studies comparing lumbar lordosis angle in people with and without LBP when standing is presented in Figure 2. Most studies reported small, non-significant differences between groups. The pooled difference (SMD = 0.01, 95% CI -0.09 to 0.11, p = 0.89) was not significant. A post-hoc meta-analysis of three studies that compared genders indicated that women had greater lordosis angles than men (SMD = 0.92, 95% CI 0.8 to 1.05, p < 0.01).

### Range of motion (ROM)

**Figure 3:**
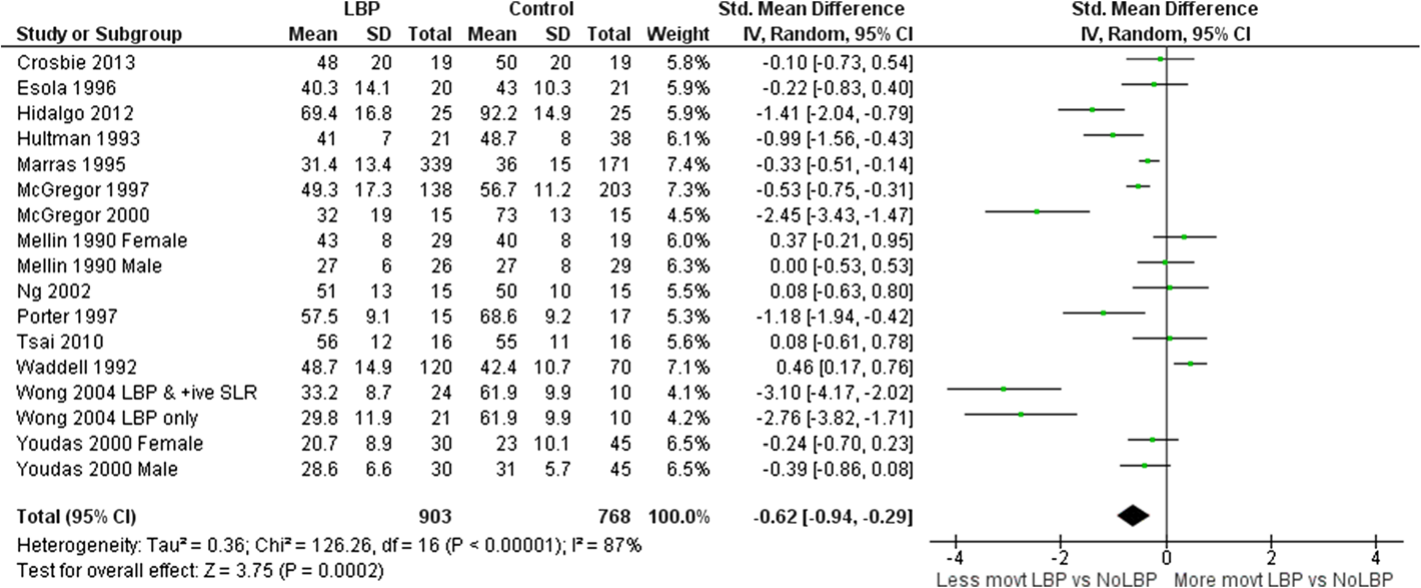
Flexion ROM meta-analysis.

**Figure 4:**
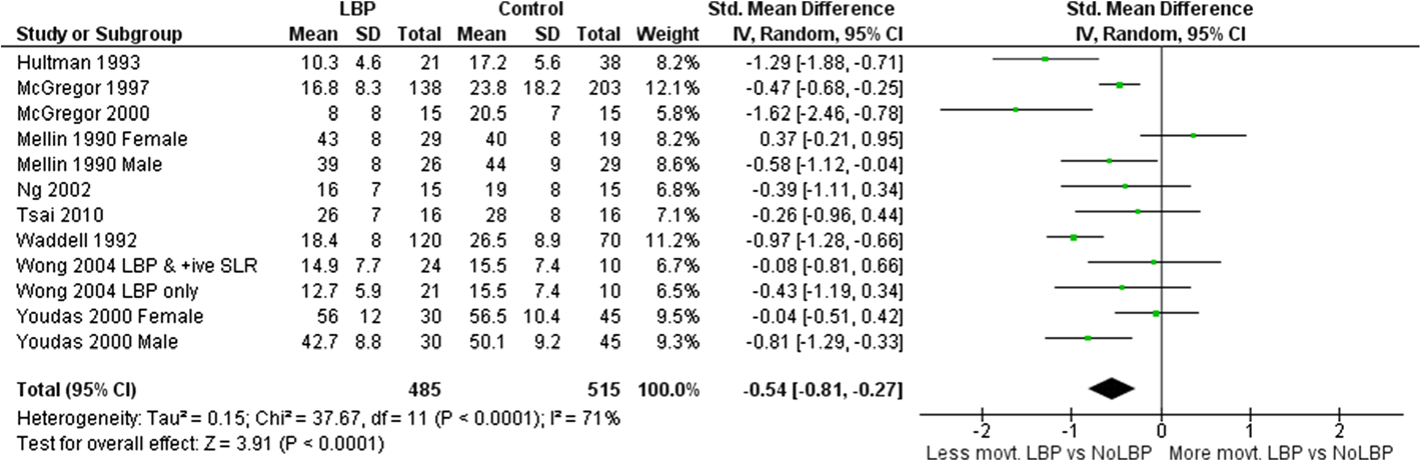
Extension ROM meta-analysis.

**Figure 5:**
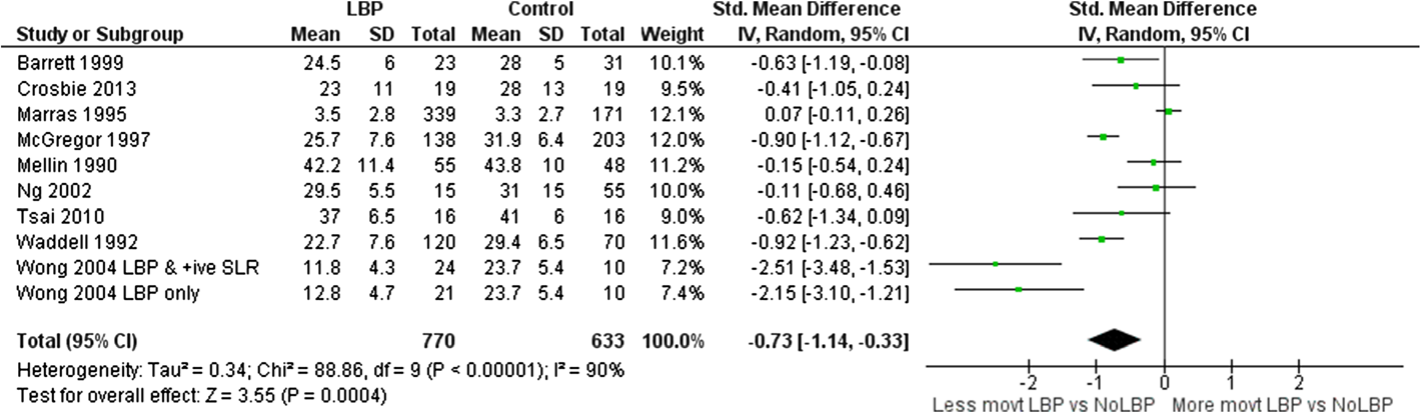
Lateral flexion ROM meta-analysis.

**Figure 6:**
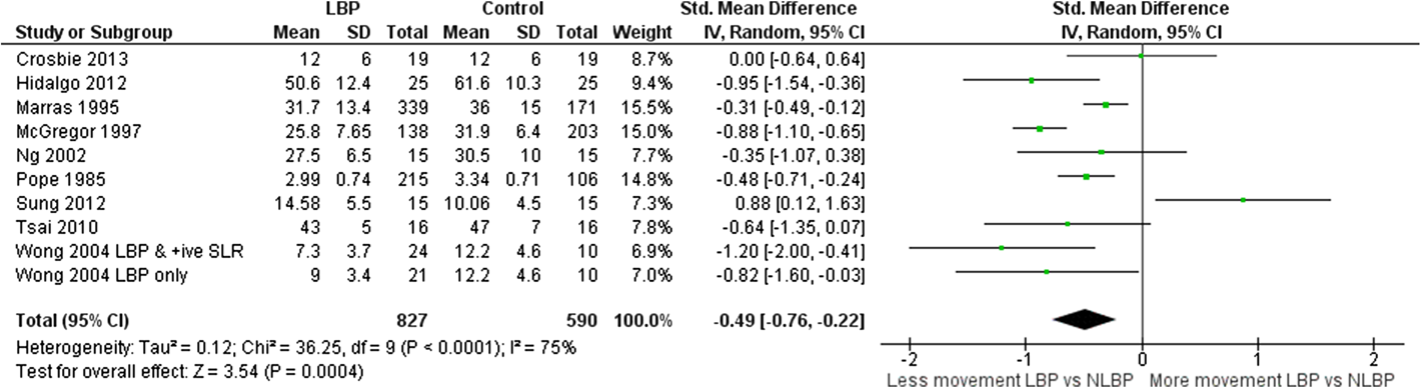
Rotation ROM meta-analysis.

Meta-analyses of 26 ROM studies consistently found reduced range of movement of the lumbar spine in people with LBP. Figures 3, 4, 5 and 6 summarise the findings for flexion, extension, lateral flexion and rotation meta-analysis. Where studies measured bilateral movement, i.e. left and right rotation, weighted means and standard deviations were averaged. In some included studies, measurements from a single group without LBP were compared with a number of LBP groups, such as men and women or acute and chronic LBP. As the observed differences may not satisfy the statistical assumption of independence required for meta-analysis [74], the sample size of these groups without LBP used in the meta-analysis were divided by the number of comparisons made. Means and standard deviations (SD) are in degrees of movement.

### Lumbar spine versus hip contribution to flexion/extension

**Figure 7:**
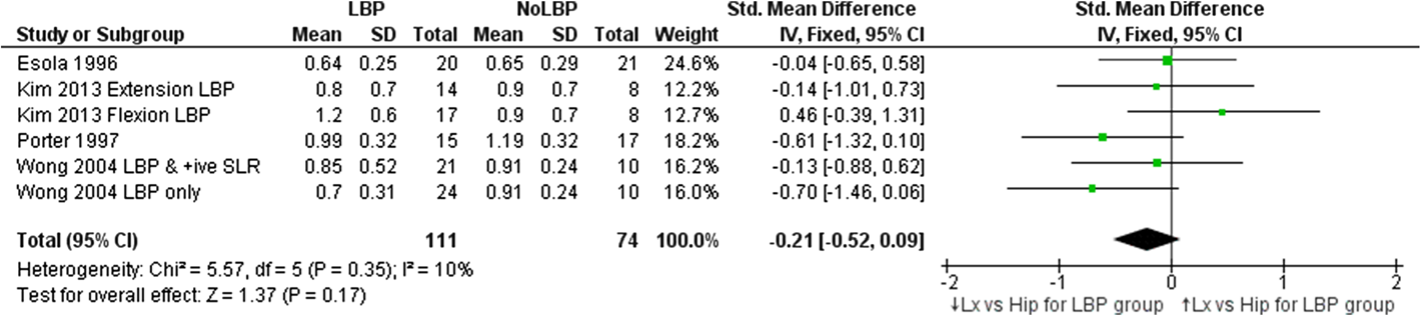
**Meta-analysis of studies investigating the relative contributions of lumbar versus hip ROM through the range of trunk flexion.** Means (and SDs) are ratios of lumbar to hip movement. Zero represents equal lumbar to hip contribution to trunk flexion, numbers <0 indicate less lumbar compared with hip movement while numbers >0 indicate more hip than lumbar movement.

Six studies examined the relative lumbar and hip contribution to flexion movements, five [34,50,52,61,71] during forward flexion, and one [40] returning from a fully flexed position. Four of five studies investigating forward flexion found no significant difference between those with and without LBP when comparing lumbar with hip contribution (ratio) to flexion ROM *at end range*. A non-significant but consistent effect favored reduced lumbar (compared with hip) contribution to flexion (Figure 7) for those with LBP (SMD = -0.21, 95% CI -0.52 to 0.09, p = 0.17). Three studies [34,40,52] found significant differences in the ‘*through-range*’ contribution of lumbar movement. Esola et al. [34] (SMD = -0.86, 95% CI -1.51 to -0.22) and Porter et al. [52] (SMD = -0.71 95% CI -1.43 to 0.00) both found significant reductions of lumbar contribution to mid-range flexion but not at end range. McClure et al. [40] found a greater contribution of the lumbar spine during mid-range return from the fully flexed position (relative extension) (SMD = 0.95 95% CI 0.10 to 1.81).

### Pelvic tilt angle, relative position and tilt range

**Figure 8:**
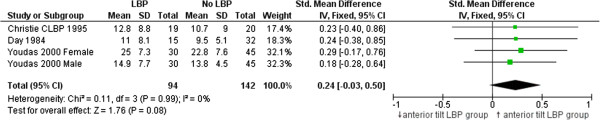
Meta-analysis of studies comparing pelvic tilt angle in neutral standing.

Three studies (four articles) examined usual pelvic tilt angle in standing [31,32,57,58]. No significant differences were found between people with or without LBP for any study (see Table 1 for details). A small, non-significant but consistent effect favouring greater anterior pelvic tilt in people with LBP was evident when studies were pooled in meta-analysis (see Figure 8). Only Day et al. [32] compared differences between groups with and without LBP in full anterior and posterior tilt positions, and found a significant difference for maximum anterior tilt angle (higher angle for people with LBP) :SMD = 0.73 (0.09 to 1.35, p = 0.02), but not maximum posterior tilt angle: SMD = 0.09 (-0.53 to 0.7, p = 0.78)).

### Speed/Acceleration

**Figure 9:**
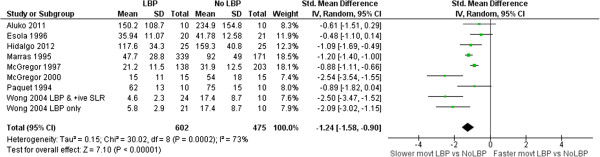
**Forest plot of speed differences between LBP and NoLBP groups (original units are deg/sec or deg/sec**^**2**^**).**

Seven studies measured speed [34,37,39,43,50,71,75] and one measured acceleration [28]. Data on lumbar flexion speed/acceleration differences between groups with and without LBP were combined in meta-analysis (Figure 9). A large, significant effect of slower movement in the LBP group was evident (SMD -1.46 95% CI -1.96 to -1.02, p < .01).

### Proprioception

**Figure 10:**
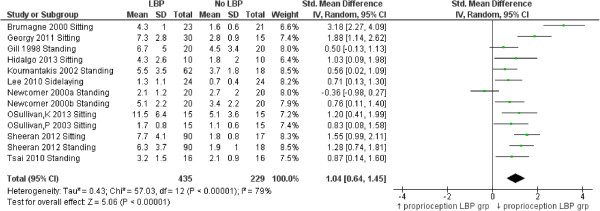
Forest plot of position/reposition differences (raw scores in degrees) comparing LBP and NoLBP groups.

Fifteen studies [33,35,45,46,53,55,60,62-68,70,76] measured position/reposition accuracy as a measure of lumbar spine proprioception (see Additional files 3, 4 and 6 for details). Twelve studies [35,45,46,53,60,62-64,68-70,76] measured absolute error in re-positioning accuracy and were included in meta-analysis. One study measured the number of trials required to achieve accurate re-positioning [33], one measured motion detection, [55] one measured ability to achieve a described position [67] and two measured motion precision [65,66] but were excluded from meta-analysis as data were not comparable. A consistent, large and significant reduction in ability to accurately re-position the spine at pre-specified angles for people with LBP compared to those without LBP is shown in Figure 10 (SMD = 1.04, 95% CI 0.64 to 1.45, p < 0.01). The studies included in this review using different types of assessments that precluded meta-analysis also found significant differences indicating reduced proprioception in the LBP group (26,55). Descarreaux et al. [33] tested if LBP subjects (divided into two groups according to normal or slow speed of force production on isometric resistance) compared to subjects without LBP, could accurately place the lumbar spine into various flexion angles. They determined that although both LBP and control groups demonstrated similar re-positioning accuracy, the LBP subgroup that developed slow isometric force (n = 9 of 16) required significantly more practice to achieve this (SMD = 1.87, 95% CI 0.89 to 2.85, p < 0.01). Taimela et al. [55] reported a significant reduction in the ability of people with chronic LBP to detect change in lumbar position when compared to a group without LBP but did not include data on variability required for meta-analysis. Field et al. [67] demonstrated reduced accuracy for people with LBP in achieving a demonstrated position in flexion when compared to people without LBP (SMD = 1.66, 95% CI 0.82 to 2.42, p < 0.01). Willigenberg et al. [65,66] also identified reduced accuracy in both motion control, (SMD = 1.14, 95% CI 0.39 to 1.89, p < 0.01) and motion tracking in people with LBP (SMD = 1.08, 95% CI 0.32 to 1.84, p < 0.01).

A summary of standardised mean differences, across all the kinematic characteristics investigated, is shown in Table 2.

**Table 2:** Summary of pooled standardized mean differences

**Position and movement differences between people with and without LBP (number of studies included in meta-analysis)**	**Standardised mean difference (95% CI) for all studies suitable for meta-analysis**
Lordosis*, n = 8	0.01 (-0.09 to 0.11), p = 0.89
Flexion**, n = 14	-0.62 (-0.94 to -0.29), p < 0.01
Extension**, n = 9	-0.54 (-0.81 to -0.27), p < 0.01
Lateral Flexion**, n = 9	-0.73 (-1.14 to -0.33), p < 0.01
Rotation**, n = 9	-0.49 (-0.76 to -0.22), p = 0.04
Lumbar versus Hip end-range flexion ROM**, n = 4	-0.21 (-0.52 to 0.09), p = 0.17
Pelvic tilt angle in standing^†^, n = 3	0.24 (-0.03 to 0.50), p = 0.08
Speed/Acceleration^‡^, n = 8	-1.24 (-1.58 to -0.90), p < 0.0001
Proprioception (re-position accuracy)^§^, n = 12	1.04 (0.64 to 1.45), p < 0.0001

*Positive numbers indicate larger lordosis for the LBP group, **negative numbers indicate reduced ROM for the LBP group, † positive numbers indicate larger anterior tilt, ^‡^negative numbers indicate reduced speed of movement for the LBP group, ^§^positive numbers indicate greater error rate in re-positioning (reduced proprioception).

### Differences in variability between groups

Table 3 presents a summary of the within group variability in movements pooled across studies. Significantly greater variability for people with LBP compared to people without LBP was observed on four of the eight measures: flexion, lateral flexion, rotation and speed/acceleration.

**Table 3:** Differences between the LBP and NoLBP in within-group variability on each movement characteristic and ratios of n-weighted mean coefficients of variation

**Movement Characteristic (number of comparisons)**	**LBP group coefficient of variation**	**N**	**NoLBP group coefficient of variation**	**n**	**Ratio of coefficients of variation (95% CI)**
Lordosis angle (8)	33.1%	818	34.6%	745	0.96 (0.83 to 1.10)
Flexion ROM* (18)	35.1%	913	26.8%	778	**1.31 (1.13 to 1.51)**
Extension ROM (12)	41.5%	485	47.2%	515	0.88 (0.76 to 1.01)
Lateral flexion ROM (9)	52.6%	751	40.1%	614	**1.31 (1.17 to 1.48)**
Rotation ROM* (10)	34.3%	827	28.7%	590	**1.20 (1.02 to 1.40)**
Lumbar vs hip (6)	51.2%	111	42.8%	74	1.2 (0.87 to 1.65)
Speed/acceleration* (8)	54.7%	602	42.6%	475	**1.28 (1.13 to 1.46)**
Proprioception (13)	53.9%	435	53.2%	229	1.01 (0.87 to 1.18)

*Statistically significant differences (95% CIs > 1.0) are bolded.

## Discussion

This review summarised the results of studies of lumbo-pelvic kinematics for people with and without LBP. Although the results will be unsurprising to most clinicians, it is the first review to meta-analyse and quantify the clinical observation that, on average, people with LBP have reduced lumbar ROM, move more slowly and have reduced proprioception compared to with those without LBP.

The review highlights the highly heterogenous nature of available studies, with six of nine meta-analyses indicating significant between study heterogeneity in results. Possible sources of heterogeneity between study outcomes include differences in definitions of back pain, control characteristics, LBP intensity, and instruments and methods for measuring movements. This heterogeneity confounds secondary analyses such as the influence of pain intensity on observed differences between people with and without LBP.

The lack of detail or standardized definition for control subjects is also problematic. For example, it is hypothetically possible that altered movement characteristics occur as a result of a LBP episode and persist after pain resolves. If this is the case, people that were pain free but with persistent altered movements, would have been eligible as control subjects for many of the included studies, provided the episode had been prior to the pain-free time period required for that study. This would have diluted differences between the groups. Similarly, it is not known if certain ‘aberrant’ movement characteristics exist prior to the onset of LBP and are risk factors for an episode of LBP, in which case these characteristics may have also been present in people classified in the included studies as control subjects.

No studies attempted to blind assessors to group type, and a general absence of procedural standardization, such as movement instruction or assessor consistency, exposes studies to the potential for random or systematic error. However, the relative consistency of the direction of results across studies adds credibility to the findings of this review, and observed effects appear large enough to be visible despite potential study limitations.

### Lordosis

Lordosis angle does not differentiate people with and without LBP. A similarly wide range of group means were reported for those with LBP (23° to 56°) and without LBP (19° to 53°). This variability might be associated with the six different measurement methods, but may also reflect biological differences in sample ethnicity [77], age [78] and gender [49,57,58]. Increasing age has been associated with reduced lordosis in the sixth decade [78-80] and on average, females have a greater lordosis than males [49,58,80]. Four studies included only males [31,32,38,47] and it is perhaps understandable that these studies found the four lowest average lordosis angles. However, this variability in lordosis appears similar for people with and without LBP. Therefore, lumbar lordosis when measured using surface techniques, does not, on average, appear to discriminate between people with and without LBP.

### Range and speed of motion

Clinicians commonly use ROM [81] to assist in identifying patterns of dysfunction, and to monitor change. ROM has been extensively studied by invasive and non-invasive methods, but non-invasive measurement is better suited to routine clinical assessment. This review included 20 studies that compared ROM for those with and without LBP using skin-surface measurement. The pooled sample was large enough to be confident in the finding that people with LBP have reduced average lumbar ROM compared to those without LBP. The mean ROM reported for people without LBP is so variable that it has little reference value e.g. (considering all studies) flexion: min = 23°, max = 92°; extension: min = 15°, max = 56°, lateral flexion: min = 3°, max = 44°; rotation: min = 3°, max = 62°. Large variations between studies suggest differences beyond those explained by biological variation and implicate method differences. Using flexion ROM as an example, 14 studies used nine different measurement devices ranging in sophistication from simple handheld inclinometers and flexible rulers to opto-electronic devices. Youdas [57,58] used a flexible rule measurement technique (mean lumbar flexion angle = 23 ± 10°) while Hidalgo [37] used an opto-electronic system (92 ± 15°); both studies used similar inclusion criteria, and the same starting position. Other method processes may also contribute to differences: two studies assessed range in sitting, 10 in relaxed standing, and two used some form of restricted movement (harness or fixed pelvic position). Based on these findings, normative data may have limited relevance to a clinical environment unless the same measurement methods used to obtain published data are also used in the clinical setting where they are applied. The lack of clarity about similarity between study populations and method details makes the use of pooled group-level estimates of movements, such as mean flexion ROM, unwise. However, these between-study differences did not obscure consistent within-study findings; eight of 14 studies of flexion demonstrated significantly less lumbar flexion for those with LBP and only one study found that lumbar flexion was significantly greater for those with LBP. These findings of large between study differences in measurements, and consistent within study differences between those with and without LBP, are similar for the other movements analysed in this review.

Lower movement speed is commonly seen in people with LBP, so it is unsurprising to observe in our review that those with LBP demonstrated significantly slower speeds when the eight included studies were pooled in meta-analysis. Reduced speed of lumbar movement has been linked to fear of movement and has also been shown to persist after recovery [82].

### Lumbar versus hip contribution to movement

Clinicians have reported assessing the relative contribution of lumbar and hip joints (during flexion and extension movements) to assist in determining subgroups within the LBP population that require specific treatment strategies [83,84]. This review identified six studies that measured patterns and relative contributions to trunk flexion from the lumbar spine and hip joints, often described as ‘lumbo-pelvic rhythm’. Data could be pooled for four studies (six comparisons) evaluating ROM of lumbar and hip contribution at end-range flexion. A typical pattern of lumbar versus hip movement for both groups showed less lumbar and greater hip ROM at end-range flexion, with small, non-significant differences of reduced lumbar contribution for the LBP group when compared to people without LBP.

However relative contributions of lumbar spine and hip to ROM may be less important than patterns of when and how movement takes place. Nelson-Wong et al. [84] recently reported that the relative timing of hip and lumbar movement when arising from a fully flexed position differentiated between people who do or do not develop back pain after two hours of standing. People who developed pain used a lumbar > hip initiation of movement (spine moves first followed by pelvic/hip movement) strategy on arising from the flexed position while non-pain developers used a hip > lumbar strategy (p = 0.03). This finding is supported by McClure et al. [40], Esola et al. [34] and Porter et al. [52] who all reported relatively greater lumbar through-range contribution in people with LBP on flexion movement. It may be that people with LBP can be subgrouped by lumbo-pelvic rhythm. For example, Kim et al. [61] examined lumbo-pelvic rhythm by comparing two subgroups of people with LBP to a group of people without LBP. One subgroup had pain provoked by flexion/rotation activities and the other by extension/rotation activity. The flexion-aggravated group had significantly greater lumbar contribution to flexion compared to the normal and extension groups. The extension-aggravated group on the other hand had a significant pattern of reduced lumbar contribution to flexion. Lumbar versus hip contributions to movement, particularly flexion, appear to have clinical relevance and warrant further exploration.

### Pelvic tilt angle, position and range

Extreme (end-range) pelvic tilt angle in standing and sitting has been linked to back pain [85,86] but with limited evidence. Clinical interventions aiming to modify pelvic tilt angle to achieve more neutral positions are based on the assumption that there is a relationship between position and pain. There are few studies that explore the relationship between LBP and typical pelvic tilt range (from full anterior to full posterior tilt) and the relative position of pelvic tilt angle during sitting and standing in people with and without LBP. This review found no differences when pooling data from three studies that compared standing pelvic tilt angle in people with and without LBP. Similarly, Astfalk et al. [85] found no differences in average lumbar flexion angle in sitting (reflecting pelvic tilt position) when comparing adolescents with and without LBP (125.3 ± 19.8° vs 130.6° ± 15.7 respectively). However significant differences were observed for lumbar flexion angle when adolescents with LBP were sub-grouped based on direction of movement that provoked pain. The flexion-provoked pain group had a significantly greater lumbar angle (135.6 ± 16.9°, p < 0.05) compared to those without LBP while the extension-provoked pain group had a significantly smaller lumbar angle (113.5 ± 16.3°, p < 0.05) when compared to those without LBP. Sub-grouping of a LBP population based on the relationship of aggravating activities and direction of painful movement may demonstrate associations between back pain and pelvic tilt angle/relative position.

### Proprioception

Our meta-analysis of studies measuring one aspect of proprioception (absolute error during re-positioning trials) demonstrated a significant and large loss of re-positioning accuracy in the LBP group. The implications of reduced proprioception are that people with LBP are less ‘movement-aware’ with potentially reduced postural control. This is consistent with a recent systematic review on another aspect of proprioception, postural sway, by Ruhe et al. [17] who found that greater sway excursion and speed were present in people with LBP compared to people without back pain.

### Differences in variability between people with and without LBP

Our assessment of differences in variability between people with and without LBP for nine movement characteristics demonstrated significantly greater variability for four movement characteristics: flexion, lateral flexion and rotation ROM, and speed of movement. There were no significant differences in variability for lordosis, extension ROM, lumbar versus hip contribution to movement or proprioception. It is not clear if the greater variability seen in the LBP group is clinically meaningful (10% difference in average variability estimates) but it raises a question of whether postures or activities performed using extremes of certain movement (e.g. excessive or restricted movement) may predispose people to LBP.

This review examined differences in group means for people with and without LBP. Given the high variability seen between studies, the small between-group differences compared with the high within-group differences, and the greater variability on some movement characteristics seen in the LBP group, these findings cast some doubt on whether an assessment of movements without reference to pain provides evidence of dysfunction at an individual patient level. The results neither endorse nor disqualify the role of movement assessment for (i) determining the relationship between movement and pain in individual patients, or (ii) monitoring changes in movement characteristics as a means of monitoring progress in individual patients and as an indication of the likelihood of their improvement [87]. Key questions also remain, including (a) are deficits such as reduced proprioception, reduced ROM and speed of movement a result or a cause of LBP, and (b) are these deficits present prior to the development of LBP?

### Strengths and limitations

The strengths of this systematic review are the comprehensive search, the breadth of the movement characteristics included in the analysis, and that screening and data extraction were independently performed by two reviewers. In addition, the review only included studies that assessed people with and without LBP using the same within-study method, thereby removing method differences as an explanation for observed within-study differences.

The review also has limitations. We treated the data for people with LBP as if they were measurements of a homogenous group. It is possible that sub-grouping by using the relationship of pain to movement may increase the clinical utility of particular measurements. The findings in this review do not inform clinicians about whether changes in ROM, movement speed or proprioception will produce better outcomes, or if changes in movement characteristics precede the onset of LBP or predispose to future recurrences. In addition, due to an absence of translation resources, only articles published in English were included and this may introduce a language, cultural and/or publication bias. To maximize the number of included studies, we did not place any restrictions on the criteria used to define pain cases versus pain-free controls. However, our broad inclusion criteria are likely to have weakened, rather than strengthened differences seen between people with and without LBP, and in the included studies, higher pain intensities had a weak correlation with increased differences between the these groups.

## Conclusion

This paper systematically summarised what is known about differences in measurements of lumbo-pelvic movement for people with and without back pain. It included 43 studies and synthesised information on six movement characteristics: lordosis, ROM, lumbar versus hip contribution, pelvic tilt, speed and proprioception. The results show that compared to people without pain, on average, people with LBP display (i) no difference in their lordosis angle (8 studies), (ii) a reduction of lumbar ROM in all directions of movement (26 studies), (iii) no difference in lumbar versus hip ROM contribution to full flexion (4 studies), (iv) no difference in pelvic tilt angle in standing (3 studies), (v) slower lumbar movement (7 studies), and (vi) poorer proprioception on position-reposition accuracy (15 studies). There is greater movement variability for people with LBP for flexion, lateral flexion and rotation ROM, and speed of movement, but this is not apparent for other movement characteristics. So put simply, when considered collectively, people with LBP have reduced lumbar ROM, move more slowly and have reduced proprioception compared with people without low back pain.

## Abbreviations

LBP: Low back pain; ROM: Range of motion; SMD: Standardised mean difference; NoLBP: People without low back pain.

## Competing interests

No funding was received for this systematic review. No benefits in any form have been, or will be, received from a commercial party related directly or indirectly to the subject of this paper. This paper does not contain information about medical devices or drugs. The authors do not hold stocks or shares in any company that might be directly or indirectly affected by this review. No patents have been applied for or received due to the content of this review. There are no non-financial competing interests associated with this review.

## Authors’ contributions

RL and JG contributed to data collection. RL and JG performed data inclusion and extraction with JK providing arbitration when required. All authors were involved in the design of the review, analysis and interpretation of data, drafting and revision of the manuscript, and gave approval of the final manuscript.

## Pre-publication history

The pre-publication history for this paper can be accessed here:

http://www.biomedcentral.com/1471-2474/15/229/prepub

## Supplementary Material

Additional file 1 Search strategy medline. Click here for file

Additional file 2 Quality assessment. Click here for file

Additional file 3 Categories of included studies. Click here for file

Additional file 4 Characteristics of included studies. Click here for file

Additional file 5 Quality assessment. Click here for file

Additional file 6 Summary of studies examining lumbar proprioception. Click here for file
