# Supplementary material for: Comparing lumbo-pelvic kinematics in people with and without back pain: a systematic review and meta-analysis
Source: BMC Musculoskelet Disord. 2014 Jul 10;15:229. doi: 10.1186/1471-2474-15-229 (PMC4096432; doi:10.1186/1471-2474-15-229)
Supplement: Additional file 1 — Search strategy medline. [file 1471-2474-15-229-S1.docx]

# Additional file 1 – Search Strategy Medline

**Sample of Medline search strategy**

| **#** | **Search Statement** | **Results** |
| --- | --- | --- |
| 1 | (Normative or normal or adult or in vivo).mp. [mp=title, abstract, original title, name of substance word, subject heading word, keyword heading word, protocol supplementary concept word, rare disease supplementary concept word, unique identifier] | 5500580 |
| 2 | (back pain or pain or lumbago or low back pain or LBP or spondylosis or lumbo-pelvic or lumbopelvic or pelvis or pelvic or vertebro-femoral or vertebrofemoral or trunk).mp. [mp=title, abstract, original title, name of substance word, subject heading word, keyword heading word, protocol supplementary concept word, rare disease supplementary concept word, unique identifier] | 555926 |
| 3 | (Skin-surface or skin or surface or surface-mounted or electronic or opto-electronic or inclinometer or inclinometry or goniometer or measurement or measurements or reliability or validity or strain gauge ORinertial or accelerometry or accelerometer).mp. [mp=title, abstract, original title, name of substance word, subject heading word, keyword heading word, protocol supplementary concept word, rare disease supplementary concept word, unique identifier] | 2140465 |
| 4 | (Movement or movements or movement pattern or movement patterns or pattern or flexibility or mobility or motion or motion analysis or lordosis or kinematic or kinematics or posture or postural or position or range of motion or range or flexion or extension or lateral flexion or sidebending or rotation or rhythm or proprioception or re-position or reposition or repositioning or re-positioning or temporal or timing or speed or velocity or acceleration or sitting or standing).mp. [mp=title, abstract, original title, name of substance word, subject heading word, keyword heading word, protocol supplementary concept word, rare disease supplementary concept word, unique identifier] | 2562142 |
| 5 | 1 and 2 and 3 and 4 | 19229 |
| 6 | limit 5 to english language | 17853 |
| 7 | limit 6 to humans | 17180 |
| 8 | (surg$ or fusion or decompression or laminectomy or discectomy or aneurysm or arter$ or fractur$ or injection$ or drug$ or pharmaceutical).mp. [mp=title, abstract, original title, name of substance word, subject heading word, keyword heading word, protocol supplementary concept word, rare disease supplementary concept word, unique identifier] | 4718892 |
| 9 | 7 not 8 | 10164 |
| 10 | (cervical or neck or ankle or knee or shoulder or elbow or hand or wrist).mp. [mp=title, abstract, original title, name of substance word, subject heading word, keyword heading word, protocol supplementary concept word, rare disease supplementary concept word, unique identifier] | 783838 |
| 11 | 9 not 10 | 6749 |
| 12 | (tumor$ or tumour$ or carcinoma or osteonecrosis or neoplasm$ or cancer$ or bone graft$).mp. [mp=title, abstract, original title, name of substance word, subject heading word, keyword heading word, protocol supplementary concept word, rare disease supplementary concept word, unique identifier] | 2651122 |
| 13 | 11 not 12 | 6215 |
| 14 | (osteoarthritis or effusion or ischiofemoral or acetabul$ or anteversion or retroversion or hip replacement or prosthe$).mp. [mp=title, abstract, original title, name of substance word, subject heading word, keyword heading word, protocol supplementary concept word, rare disease supplementary concept word, unique identifier] | 312593 |
| 15 | 13 not 14 | 5951 |
| 16 | (metabol$ or osteoporo$ or osteopen$ or aneurysm or injection$ or fusion or urinary).mp. [mp=title, abstract, original title, name of substance word, subject heading word, keyword heading word, protocol supplementary concept word, rare disease supplementary concept word, unique identifier] | 2079392 |
| 17 | 15 not 16 | 5707 |
| 18 | (urinary or rect$ or kidney or renal or nephro$ or pudendal).mp. [mp=title, abstract, original title, name of substance word, subject heading word, keyword heading word, protocol supplementary concept word, rare disease supplementary concept word, unique identifier] | 1215521 |
| 19 | 17 not 18 | 5437 |
| 20 | (scoliosis or scoliotic or idiopathic).mp. [mp=title, abstract, original title, name of substance word, subject heading word, keyword heading word, protocol supplementary concept word, rare disease supplementary concept word, unique identifier] | 94643 |
| 21 | 19 not 20 | 5324 |
