# Supplementary material for: Comparing lumbo-pelvic kinematics in people with and without back pain: a systematic review and meta-analysis
Source: BMC Musculoskelet Disord. 2014 Jul 10;15:229. doi: 10.1186/1471-2474-15-229 (PMC4096432; doi:10.1186/1471-2474-15-229)
Supplement: Additional file 2 — Quality assessment. [file 1471-2474-15-229-S2.docx]

# Additional file 2: Quality Assessment

## Criteria definition and decision rules

|  | **Criteria** | **Decision rule** |
| --- | --- | --- |
| **Selection bias** | | |
|  | Was the study population adequately described? | Age (mean+SD, or range), sex (male vs female subjects), BMI (or weight) ±SD). All three variables much be included to score yes. |
|  | Where both groups drawn from the same population? | People were from the same setting, eg people with and without back pain from a single setting such as a university, OR were they matched age/sex/BMI or weight case controls. If so score yes, if no or no data, score no. |
|  | Were both groups comparable for age, sex, BMI/weight | Was a comparison made between groups on these parameters? Yes if comparison made AND groups were comparable. No if not comparable or no comparison made. |
|  | Was pain intensity and/or activity limitation described for LBP group? | Score yes if measured using a validated scale, such as a Visual Analogue Scale or Numeric Rating Scale for pain or the Oswestry Disability Index or Roland Morris Disability Questionnaire or similar. |
|  | Was an attempt made to define back pain characteristics? | (i) Stage (acute/subacute/chronic) (ii) +/- leg pain? (iii) Information on specific vs non-specific diagnosis. Score yes if at least two out of these three variables covered. |
| **Measurement and outcome bias** | | |
|  | Did the method description enable accurate replication of the measurement procedures | Description enables accurate replication of the measurement procedures (score yes). |
|  | Was the measurement instrument adequately described? | Instrument used to measure described (score yes). |
|  | Was a system for standardizing movement instructions reported? | A system for standardizing movement instructions is reported (score yes). |
|  | Were assessors trained in standardized measurement procedure? | Yes if report of training, or no, if no mention of training process. |
|  | Did the same assessors test those with and without back pain | If yes then score yes. If no detail score no. |
|  | Were assessors blinded as to which group subjects were in? | If blinding attempted, was it evaluated and found to be successful (e.g. attempting to guess group assignment resulted in answers that could occur by chance alone). |
|  | Was the same assessment procedure applied to those with and without back pain? | If there was any difference to procedure or measurement then score no. |
| **Data presentation** | | |
|  | *Statistical analysis bias*  The results of between-group statistical comparisons are reported for at least one key outcome | Yes or no. |
|  | Point estimates and measures of variability are provided for at least one key outcome for those with and without back pain | Yes or no. |

A “no” score indicates that no data or information was provided.
