# Supplementary material for: Comparing lumbo-pelvic kinematics in people with and without back pain: a systematic review and meta-analysis
Source: BMC Musculoskelet Disord. 2014 Jul 10;15:229. doi: 10.1186/1471-2474-15-229 (PMC4096432; doi:10.1186/1471-2474-15-229)
Supplement: Additional file 3 — Categories of included studies. [file 1471-2474-15-229-S3.docx]

# Additional file 3: Categories of included studies (✓ = included, ✓✓ = appropriate for meta-analysis)

|  | **Author, Date** | **Lordosis in standing** | **Lumbar spine ROM**  **(standing unless otherwise stated)** | | | | **Pelvic tilt position or ROM** | **Hip versus lumbar contribution** | **Velocity/**  **Acceleration** | **Proprioception**  **re-position accuracy** | **Data appropriate for meta-analysis**  **(or reasons for not including)** |
| --- | --- | --- | --- | --- | --- | --- | --- | --- | --- | --- | --- |
|  |  |  | F | E | LF | Rot |  |  |  |  |  |
|  | Aluko, 2011 |  |  |  |  |  |  |  | **✓✓**  F, E in standing |  | **✓✓** |
|  | Barrett, 1999 |  |  |  | **✓✓** |  |  |  |  |  | **✓✓** |
|  | Boline, 1992 |  |  |  |  | ✓ (in 90^o^ F) |  |  |  |  | position too different |
|  | Brumagne  2000 |  |  |  |  |  |  |  |  | **✓✓** sitting | **✓✓** |
|  | Christie, 1995 | **✓✓** |  |  |  |  | ✓sitting, standing |  |  |  | **✓✓** |
|  | Crosbie, 2013 |  | **✓✓** |  | **✓✓** | **✓✓**  sitting |  |  |  |  | **✓✓** |
|  | Day, 1984 | **✓✓** |  |  |  |  | ✓ supine, standing |  |  |  | **✓✓** |
|  | Descarreaux, 2005 |  |  |  |  |  |  |  |  | ✓ standing | different method of measuring proprioceptiion |
|  | Esola, 1996 |  | **✓✓** |  |  |  |  | **✓✓** | **✓✓** F |  | **✓✓** |
|  | Field, 1997 |  |  |  |  |  |  |  |  | ✓ standing | only study to use positioning rather than re-positioning approach |
|  | Georgy, 2011 |  |  |  |  |  |  |  |  | **✓✓** sitting | **✓✓** |
|  | Gill, 1998 |  |  |  |  |  |  |  |  | **✓✓** standing, 4 point kneeling | **✓✓** |
|  | Gomez, 1994 |  |  |  | ✓ | ✓ |  |  |  |  | compared asymmetries but not ROM |
|  | Hidalgo, 2012 |  | **✓✓** |  |  | **✓✓** |  |  | **✓✓** F, Rot |  | **✓✓** |
|  | Hildago, 2013 |  |  |  |  |  |  |  |  | **✓✓** sitting | **✓✓** |
|  | Hultman, 1992 | **✓✓** | **✓✓** | **✓✓** |  |  |  |  |  |  | **✓✓** |
|  | Kim, 2013 |  |  |  |  |  |  | **✓✓** |  |  | **✓✓** |
|  | Lee, 2010 |  |  |  |  |  |  |  |  | **✓✓**Sitting, sidelying, supine | **✓✓** |
|  | Koumantakis, 2002 |  |  |  |  |  |  |  |  | **✓✓** standing | **✓✓** |
|  | Marras, 1995 |  | **✓✓** |  | **✓✓** | **✓✓** |  |  | **✓✓** F, E, LF, Rot |  | **✓✓** |
|  | McClure, 1997 |  |  |  |  |  |  | ✓ |  |  | only study on return from F |
|  | McGregor, 1995,1997 |  | **✓✓** | **✓✓** | **✓✓** | **✓✓** |  |  | **✓✓** F,E, LF, Rot |  | **✓✓** |
|  | McGregor 2000 |  | **✓✓** | **✓✓** |  |  |  |  | F,E |  | **✓✓** |
|  | Mellin 1990 |  | **✓✓** (sitting) | **✓✓**  (4 point kneeling) | **✓✓** |  |  |  | F (sitting),E (4 point kneeling) |  | **✓✓** |
|  | Newcomer, 2000A |  |  |  |  |  |  |  |  | **✓✓** standing | **✓✓** |
|  | Newcomer, 2000B |  |  |  |  |  |  |  |  | **✓✓** standing | **✓✓** |
|  | Ng, 2002 | **✓✓** | **✓✓** | **✓✓** | **✓✓** | **✓✓** |  |  |  |  | **✓✓** |
|  | Norton, 2004 | **✓✓** |  |  |  |  |  |  |  |  | **✓✓** |
|  | Nourbakhsh, 2001 | **✓✓** |  |  |  |  |  |  |  |  | **✓✓** |
|  | O’Sullivan, 2003 |  |  |  |  |  |  |  |  | **✓✓** sitting | **✓✓** |
|  | O’Sullivan, 2013 |  |  |  |  |  |  |  |  | **✓✓** sitting | **✓✓** |
|  | Paquet, 1994 |  | ✓ |  |  |  |  | ✓ | **✓✓** F |  | **✓✓**  ✓measured from T8 (all others measured from T12) |
|  | Pope, 1985 |  | ✓ | ✓ | ✓ | **✓✓** |  |  |  |  | **✓✓**  ROM measurement units possibly not comparable |
|  | Porter, 1997 |  | **✓✓** |  |  |  |  | **✓✓** |  |  | **✓✓** |
|  | Sheeran, 2012 |  |  |  |  |  |  |  |  | **✓✓** standing, sitting | **✓✓** |
|  | Sung, 2012 |  |  |  |  | **✓✓** |  |  |  |  | **✓✓** |
|  | Taimela, 1999 |  |  |  |  |  |  |  |  | ✓ sitting | only study on motion detection |
|  | Tsai, 2010 |  | **✓✓** | **✓✓** | **✓✓** | **✓✓** |  |  |  | **✓✓** standing | **✓✓** |
|  | Waddell, 1992 | **✓✓** | **✓✓** | **✓✓** | **✓✓** |  |  |  |  |  | **✓✓** |
|  | Willigenburg, 2012 |  |  |  |  |  |  |  |  | ✓ kneelsitting | only study on motion control precision |
|  | Willigenburg, 2013 |  |  |  |  |  |  |  |  | ✓ kneelsitting | only study on motion tracking precision |
|  | Wong, 2004 |  | **✓✓** | **✓✓** | **✓✓** | **✓✓** |  | **✓✓** | **✓✓** F, E, LF , Rot |  | **✓✓** |
|  | Youdas, 1996, 2000 | **✓✓** | **✓✓** (sitting) | **✓✓** (prone) |  |  | ✓ |  |  |  | **✓✓** |
