# Supplementary material for: Comparing lumbo-pelvic kinematics in people with and without back pain: a systematic review and meta-analysis
Source: BMC Musculoskelet Disord. 2014 Jul 10;15:229. doi: 10.1186/1471-2474-15-229 (PMC4096432; doi:10.1186/1471-2474-15-229)
Supplement: Additional file 4 — Characteristics of included studies. [file 1471-2474-15-229-S4.docx]

# Additional file 4: Characteristics of included studies

## All studies listed alphabetically

| **Study (1^st^ author), Date** | **n,**  **sex,**  **age** | **BMI**  **or**  ***Weight*** *(kg)* | **Sample source**  *(and NoLBP definition)* | **Back pain definition**   - Duration - Type: (symptom pattern, diagnosis, +/- leg pain), - Pain/Activity limitation: | **Back pain at time of testing? (yes/no)** | **Level of pain**  **Low=0-20%**  **Med=21-50%**  **High=>50%** | **Measurement**  **device and region**  **measured** | **Movement kinematics measured** | **Movement method & instructions** |
| --- | --- | --- | --- | --- | --- | --- | --- | --- | --- |
| Aluko,  2011 | 10 NoLBP  10 LBP  Male (M) & Female (F)  21-51 years | ***NoLBP:*** *71±14 kg*  ***LBP:***  *74±21* | ***NoLBP:*** University staff  *No LBP for previous 6 months*  ***LBP****:* Local hospital patients | ***Duration:*** 5.7±1 weeks  ***Type:*** non-specific LBP  ***Pain/Activity limitation::***  VAS 29±23%  RMDQ 32±27% | **Yes** | Medium | Lumbar Motion monitor  ***Region:*** T12-S2 | Flexion, extension speed from neutral standing,  (velocity: m/sec) | “as many flexion/extension repetitions as possible in 8 seconds” |
| Barrett  1999 | 31 NoLBP  (M:14, F:17)  20-34 years  LBP  (M:13, F:10)  19-33 years | 🗶 | ***NoLBP:*** subjects recruited from local advertising  *No LBP for previous 6 months & no history of surgery* ***LBP:*** sourced from private physiotherapy practices | ***Duration:*** unknown  ***Type:*** +/- leg pain + stiffness on movement assessment, excluded subjects with BMI> 30  ***Pain/Activity limitation:*** not stated | **Yes** | not stated | Electro-goniometer (3Space Fastrak, Polhemus)  ***Region:*** L3-S1 | Lateral flexion in neutral, flexion and extension (^o^) | Neutral standing, flexion to pain onset (or pain increase if pain in neutral standing) 3 repetitions, flexion with lateral flexion added at end range flexion (left then right),similarly for extension + lateral flexion (1 repetition) |
| Boline, 1992 | 25 NoLBP  (M:8, F:17)  25 LBP  (M:14, F:11)  28-38 years | 🗶 | ***NoLBP****:* University staff and students  *No “recent LBP lasting >2 weeks”, no history of surgery*  ***LBP:*** patients from outpatient clinic of University | ***Duration:*** back pain > 6 months  ***Type:*** not stated  ***Pain/Activity limitation:*** not stated | **Yes** | not stated | Inclinometer  ***Region:*** T12/L1-pelvis | Rotation in 90^o^ flexion (^o^) | “Brief warm up period”  One repetition |
| Brumagne  2000 | 21 NoLBP  (M:6, F:15)  22.3±3.8  23 LBP  (M:7, F:16)  21.8±2.1 | ***NoLBP:*** *63.2±7 kg*  ***LBP:*** *64.9±7.2* | ***NoLBP:*** University students or staff  *no definition for NoLBP*  ***LBP:*** Hospital outpatient departments | ***Duration:*** not stated  ***Type:*** not stated  ***Pain/Activity limitation:***  VAS 38*±*17%  ODI 14*±*13.6% | **Yes** | low | Electrogoniometer (accelerometer)  ***Region:*** S2 | Pelvic tilt re-position error in ***sitting*** (constant, variable and absolute error) | Measured sacral (pelvic) tilt angle in sitting, ROM of pelvic tilt. Criterion angle determined, held 5 seconds, then full anterior tilt followed by a return to perceived criterion position. Repeated 5 times. |
| Christie  1995 | 20 NoLBP  39 LBP  M&F  18-46 years | ***NoLBP:*** 22.8±2.3  ***LBP:*** (acute) 25.9±4.2 (chronic) 24.7±3.3 | ***NoLBP:*** *no LBP for previous 12 months and never pain for > 1 month*  ***NoLBP & LBP:*** subjects sourced from selected medical institutions & university campus | ***Duration:*** Two groups of LBP:  Group 1 > 6 months  Group 2 < 6 months  ***Type:*** not stated  ***Pain/Activity limitation:*** not stated | **Yes** | not stated | Photography  ***Region:*** T12-L5 (lordosis)  ASIS-PSIS (pelvic tilt angle) | Lordosis in standing, sitting (°)  Pelvic tilt in standing – neutral position (° angle from horizontal) | Instructions only described for postural assessment:  Relaxed standing  Standardised sitting instruction |
| Crosbie  2013 | 19 NoLBP  (M:6, F:13)  28.6±5.4  19 LBP  (M:7, F:12)  34.0±13.3 | ***NoLBP:*** 23.0±2.4  ***CLBP:***  24.5±3.6 | ***NoLBP & LBP:***  No detail provided | ***Duration:*** 72month (range 8-150month) recurrent episodes  ***Type:*** not stated  ***Pain/Activity limitation::***  VAS 29±23%  RMDQ 21% (range 0-79%) | **Yes** | *low* | Electro-magnetic  (Motion Star wireless)  ***Region:*** T6 -L1, L1-S2 (low thoracic and lumbar regions) | Flexion, rotation, lateral flexion, (^o^) & in walking | “several” flexion, lateral flexion movements in standing, axial rotation in sitting |
| Desscareaux 2005 (and 2004) | 15 NoLBP  (M:9, F:6)  38.2 years  16 LBP  (M:11, F:5)  41.5 years | ***NoLBP:***  *71.9±12.1 kg*  ***LBP:***  *Group 1: 75.4±13.9*  *Group 2:*  *68.1±15.2* | ***NoLBP:*** “local advertising” *No definition*  ***NoLBP & LBP:*** “local advertising” | ***Duration:*** not stated  ***Type:*** not stated  ***Pain/Activity limitation:*** LBP group 1 (longer time to produce isometric force,  VAS 14±8%, ODI 27.4±10.8%  LBP group 2 (shorter time to produce isometric force)  VAS 43±9%  ODI 26±9.3% | **Yes** | *low and medium* | “biomedical device” (Loredan Biomedical) – no other detail provided | ***Standing*** flexion (15^o^, 30^o^, 60^o^), extension (15^o^) reposition accuracy | Neutral standing with pelvis & legs immobilised, then flexion to 15°, 30° & 60°, extension to 15°. 1 repetition performed then visual feedback training until accurate repositioning to within 10% of predetermined position. Data collected from 10 consecutive trials without feedback |
| Day  1984 | 32 NoLBP  (M:32)  15 LBP  (M:25)  25-55 years | 🗶 | ***NoLBP:*** limited detail,  *No LBP for previous 6 months & no history of surgery* ***LBP:*** subjects from orthopaedic clinic | ***Duration:*** not stated  ***Type:*** not stated  ***Pain/Activity limitation:*** not stated | **Yes** | not stated | Electro-mechanical (Iowa anatomical position systems)  ***Region:*** ASIS-PSIS, T12-S2 | Lordosis (depth of curve- mm)  Pelvic tilt – neutral (angle from horizontal), full anterior, full posterior (^o^) | Supine & standing pelvic tilt, practice followed by 3 recorded repetitions |
| Esola,  1996 | 21 NoLBP  (M:13, F:8)  23-37 years  20 LBP  (M:14, F:6)  23-46 years | ***NoLBP:***  *71.9±12 kg*  ***LBP:***  *75.2±17* | ***NoLBP & LBP:***  No detail provided | ***Duration:*** LBP episode within last 5 years. No pain at time of testing. Average time since last episode (episode not defined) = 12.8±16.9 months  ***Type:*** not stated  ***Pain/Activity limitation:*** ODI 23.6±18 (at last episode)  VAS 34.2±23.8 (at last episode) | **No** | *NA* | Opto-electronic (Watsmart) 3D motion analysis system  ***Region:*** T12-S2 | Flexion of lumbar spine versus hip (^o^) & (velocity: m/sec) | Relaxed, standing position, 3 practice flexion movements, 3 recorded movements at self-selected velocity, 15 sec rest between movements |
| Field  1997 | 16 NoLBP  (M:16)  37.9 ±7.4 years  16 LBP  (M:16)  38.3 ±8 years | ***NoLBP:***  *83±8.8 kg*  ***LBP:***  *87±17.4* | ***NoLBP:*** no detail  *No definition*  ***LBP:*** subjects from outpatient hospital clinic | ***Duration:*** > 4 months  ***Type:*** no detail  ***Pain/Activity limitation:***  no detail | **Yes** | not stated | Electro-goniometer (Orthoranger II)  ***Region:*** trunk angle at L3 | Positioning error (o) | Relaxed standing, subjects shown a diagram of 45otrunk flexion (target position), blindfolded then asked to assume target position, 4 repetitions, then 4 repetitions holding 5kg load |
| Georgy  2011 | 15 NoLBP  38.5±5.9 years  30 LBP  39.9±5.3 years | ***NoLBP:***  *83.3±8.8 kg*  ***LBP:***  *83±11.9* | ***NoLBP:*** relatives and friends of patients,  *No definition*  ***LBP:*** subjects from outpatient hospital clinic | ***Duration:*** > 3 months  ***Type:*** Non specific and “discogenic” diagnosis (combined)  ***Pain/Activity limitation:***  ODI 30.7±7.1  VAS 64±8 | **yes** | *high* | Isokinetic dynamometer (Biodex system3)  ***Region:*** T1-S2 | Repositioning error (absolute error) of the thoracolumbar spine (^o^) | Sitting, 3 practice runs of flexing from neutral sitting to 30o flexion, (target) then 3 tests with subject pressing button on achieving perceived target. |
| Gill  1998 | 20 NoLBP  (M:7, F:13)  24-53 years  20 LBP  (M:7, F:13)  21-74 years | 🗶 | ***NoLBP:*** subjects from hospital staff,  *No LBP that required time off work, no current pain*  ***LBP:*** subjects from outpatient hospital clinic | ***Duration:*** > 12 months  ***Type:*** +/– leg pain  ***Pain/Activity limitation:*** not stated | **yes** | not stated | Lumbar motion monitor  ***Region:*** T12-S1 | Proprioceptive position accuracy of position/reposition at 20^o^ flexion in ***standing*** & ***4-point kneeling*** | Standing (pelvis immobilised) and 4-point kneeling with visual (computer) feedback, flexion to 20^o^. 10 practice repetitions in each position. Blindfolded subjects attempted to reproduce position 10 times in 30 seconds |
| Gomez,  1994 | 168 NoLBP  (M:85, F:83)  120 LBP  (M:110, F:10)  18-68 years | ***NoLBP:***  M: *76.4±10.5 kg*  F: *59±8*  ***LBP***  M: *79.7±11.9*  F: *70.4±13.4* | ***NoLBP:*** subjects from administrative offices  *No LBP for previous 6 months & no history of surgery*  ***LBP:*** subjects from workers compensation and backcare programs | ***Duration:*** Subacute and chronic (no details)  ***Type:*** not stated  ***Pain/Activity limitation:*** not stated | **Yes** | not stated | Standing dynamometer (B200 Isostation)  ***Region:*** Lumbar spine, detail not reported | Rotation & lateral flexion  Standing, strapped into B200 device  (symmetry: ratio) | “Standardised protocol” as per B200 manual (detail not provided)  Best of 2 repetitions for maximal rotation & lateral flexion  4 repetitions for flexion & extension. Reported coefficient of variation |
| Hidalgo,  2012 | 25 NoLBP  (M:10, F:15)  35 LBP  (M:12, F:13)  35-65 years | ***NoLBP:*** 23.3±2.5  ***LBP:***  25.2±3.2 | ***NoLBP:*** volunteers  *No LBP for previous 6 months & no history of surgery*  ***LBP:*** recruited from hospital program | ***Duration:*** Chronic (> 3months)  ***Type:*** No pain below knee  Non-specific LBP  ***Pain/Activity limitation:***  VAS 24±17 | **Yes** | *medium* | Opto-electronic (Elite)  ***Region:*** T12-S2 | Flexion, rotation, flexion+30^o^ rotation in seated position (^o^) & (velocity: m/sec) | 15 repetitions (10 recorded). Begin & end each movement in neutral sitting, move at self-selected speed and move as far as possible |
| Hidalgo  2013 | 10 NoLBP  (M:5, F:5)  30.0 ±11.7 years  10 LBP  (M:5, F:5)  33.8±7.5 years | ***NoLBP:*** 22.9±2.2  ***LBP:***  22.4±2.9 | ***NoLBP:*** *No details*  ***LBP:*** recruited from hospital program | ***Duration:*** 11.4±4.7 months  ***Type:*** Non-specific LBP  No pain below knee  ***Pain/Activity limitation:***  VAS 34±9 | **yes** | *medium* | Opto-electronic (Elite)  ***Region:*** T12-S2 | Repositioning error (absolute error) of the lumbar spine (^o^) | Subjects were in a seated position with corrected spine posture then maintained curvature, moving at their own pace to target position of 30° with eyes closed. One warm-up trial pausing for 3 seconds to remember position then 10 repetitions recorded |
| Hultman, 1993 | 38 NoLBP  (M:38)  50.2±3 years  21 LBP  (M:21)  48.6±5.7 years | ***NoLBP:*** 26.3±4.3  ***LBP:***  26.4±4.3 | ***NoLBP (group1):*** Workers from industrial company,  *No significant LBP ever*  ***LBP (group 3):*** Workers & patients referred to hospital outpatient department | ***Duration:*** Chronic (>3 years and 3 months of work)  ***Type:*** not stated  ***Pain/Activity limitation:*** not stated | **Yes** | not stated | Debrunners kyphometer  ***Region:*** T12-S2 | Lordosis (angle)  Flexion, extension (^o^) | Relaxed standing  One practice movement then two recorded movements  Standardised instructions |
| Kim  2013 | 16 NoLBP  23.8±2.9 years  17 LBP (flexion)  23.5±2.4 years  14 LBP (extension)  23.8±3.9 years | ***NoLBP:***  *61.3±9.2 kg*  ***LBP flexgrp:***  *67.2±11.9*  ***LBP ext grp:***  *65±11.2* | ***NoLBP:*** source not stated, *No definition provided*  ***LBP:*** source not stated | ***Duration:*** not stated  ***Type:*** LBP with no radiating pain  ***Pain/Activity limitation:*** not stated | **?** | not stated | Opto-electronic (Vicon) motion analysis system  ***Region:*** T12-S2 | Flexion and return (standing) of lumbar spine and hip joint (^o^)  Flexion relaxation response | Flexion from standing position, holding fully flexed position for 3 seconds. 3 trials recorded |
| Koumantakis  2002 | 18 NoLBP  (M:8, F:10)  24.6±4 years  62 LBP  (M:30, F:32)  38.2±10.7 years | ***NoLBP:***  24±2.8  ***LBP:***  26.4±3.7 | ***NoLBP:*** source not stated, *No previous LBP history*  ***LBP:*** source not stated | ***Duration:*** recurrent LBP (at least 2 episodes in last year) or > 6 weeks after acute onset  ***Type:*** mechanical non-specific LBP  ***Pain/Activity limitation:***  VAS 34.7±23.7%  RMDQ 42±35% | **Yes** | *medium* | Triaxial electro-goniometer (Lumbar Motion monitor)  ***Region:*** thoraco-lumbar spine (T12 to S2) | Repositioning error (absolute & variable error) of the lumbar spine (^o^)  For:  20^o^ flexion  15^o^ rotation  15^o^ lateral flexion | Standing unrestrained, practice of the 5 test positions (no of practice reps not recorded), then 3 repetitions to each of the 5 targets at subjects preferred speed  NB thighs touched couch to limit lower limb contribution to rotation |
| Lee  2010 | 24 NoLBP  (M:14, F:10)  42.4±9.0 years  24 LBP  (M:11, F:13)  42.6±13.7 years | ***NoLBP:***  *73±14.8 kg*  ***LBP:***  *71.3±12.8* | ***NoLBP:*** source not stated, *No definition provided*  ***LBP:*** source not stated | ***Duration:*** > 3 months  ***Type:*** No definition provided  ***Pain/Activity limitation:***  ODI 19±15%  VAS 40±26% | **yes** | *low* | Specifically made device  ***Region:*** Thoracolumbar (not clearly stated) | Repositioning error (absolute error) of the lumbar spine (^o^)  Motion perception threshold (^o^) | Seated, (axial rotation), sidelying (Flexion/extension) and supine (lateral flexion) test positions. Repositioning occurred with upper body fixed, lower trunk moving from 15o away from neutral. Subjects pressed a button when neutral position was re-achieved. 2 practice trials and 4 test trials for each test |
| Marras, 1995 | 339 NoLBP  (M:193, F:146)  171LBP  (M:96, F:75) | 🗶 | ***NoLBP:*** source not stated, *No LBP ever*  ***LBP:*** subjects from secondary & tertiary referral sources | ***Duration:*** > 7 weeks  ***Type:*** LBP+proximal radiation (n=16), LBP+distal radiation (n=17), LBP only (n=17), Listhesis (n=16), disc prolapse, pain<3 (n=12), disc prolapse, pain>3 (n=30), stenosis (n=11), nonorganic (n=17), scoliosis (n=9)  ***Pain/Activity limitation:***  not stated | **Yes** | *mixed*  *(did compare low pain to moderate + high pain subgroups for herniated disc category)* | Triaxial electro-goniometer (Lumbar Motion monitor)  ***Region:*** “primarily the lumbar spine” no other detail provided | Flexion, extension in 0^o^, 15^o^, 30^o^ of axial rotation, lateral flexion, rotation (^o^) & (velocity: m/sec) & (acceleration: m/sec^2^) | Free neutral standing, one warm up practice movement followed by 4 recorded (averaged) repetitions, standardised instruction |
| McClure (and Esola), 1997 | 12 NoLBP  23-35 years  12 LBP  23-46 years | ***NoLBP:***  *69.5±11 kg*  ***LBP:***  *78.9±15.7* | ***NoLBP & LBP:***  No detail provided | ***Duration:*** LBP episode within last 5 years. No pain at time of testing. Average time since last episode (episode not defined) = 12.8±16.9 months  ***Type:*** not stated  ***Pain/Activity limitation:***  ODI 25.7±6.9 (for last episode)  VAS 30.8±8.2 | **No** | *NA* | Opto-electronic (Watsmart) 3D motion analysis system  ***Region:*** T12-S2 | Extension (on return from full flexion) of lumbar spine versus hip (^o^) & (velocity: m/sec) | 3 practice flexion movements, 3 movements recorded at self-selected velocity, 15 sec rest between movements |
| McGregor, 1995,1997 | 203 NoLBP  (M:103, F:100)  138 LBP  (M:76, F:62) | 🗶 | ***NoLBP:*** source not stated, *No LBP for previous 6 months*  ***LBP:*** subjects from hospital outpatient clinic | ***Duration:*** not stated  ***Type:*** Non-specific LBP (n=25), disc prolapse (n=33), degen disc disease (n=57), spondylolisthesis (n=12), stenosis (n=11)  ***Pain/Activity limitation:***  VAS 51±28 | **Yes** | *high* | CA-6000 (3D potentiometer)  ***Region:*** T12-L5 | Flexion, extension, rotation, lateral flexion, (^o^) & (velocity: m/sec) | Free neutral standing, one warm up practice movement 3 repetitions averaged |
| McGregor, 2000 | 15 NoLBP  33.5 *±* 6.3 years,  15 LBP  58*±*16.4 years | 🗶 | ***NoLBP:*** staff of medical teaching college,  *“No current or recent history of LBP”*  ***LBP:*** hospital spinal clinic | ***Duration:*** not stated  ***Type:*** lumbar canal stenosis  ***Pain/Activity limitation:*** not stated | **Yes** | not stated | CA-6000 (3D potentiometer)  ***Region:*** T12-L5 | Flexion, extension, (^o^) & (velocity: m/sec) | Free neutral standing, one warm up practice movement. 3 repetitions averaged, repeated at 3 speeds: slow, preferred, fast |
| Mellin  1990 | 48 NoLBP  (M:29, F:19)  55 LBP  (M:26, F:29)  21.4±1.6 years | 🗶 | ***NoLBP & LBP:*** Nursing and medical students,  ***NoLBP:*** *no LBP in previous year* | ***Duration:*** not stated  ***Type:*** not stated  ***Pain/Activity limitation:*** not stated | **Mixed** | not stated | Inclinometer  ***Region:*** PSIS (S2) to 20cm cranial (lumbar spine) | Flexion, extension, lateral flexion, (^o^) | Flexion (in sitting), extension (in 4-point kneeling), lateral flexion (in standing) |
| Newcomer, 2000A | 20 NoLBP  (M:7, F:13)  39.1±11.3 years  20 LBP  (M:8, F:12)  39.3±11.4 years | 🗶 | ***NoLBP:*** ***:*** subjects from advertising,  *No LBP >3 months or at any time in previous year*  ***LBP:*** subjects from advertising | ***Duration:*** >3 months  ***Type:*** not stated  ***Pain/Activity limitation:***  VAS 48±18 | **Yes** | *medium* | 3Space tracker (electro-magnetic)  ***Region:*** L1 and S1 | Proprioceptive position accuracy:  flexion, extension, lateral flexion & rotation in standing (reposition error ^o^) | Relaxed neutral standing (pelvis free). Slow movement (5 seconds) flexion, extension & lateral flexion to 50% of maximum ROM. 3 repeated measures of return to 50% position were recorded. Performed with eyes open then repeated with eyes closed |
| Newcomer, 2000B | 20 NoLBP  (M:9, F:11)  39.8±12.7 years  20 LBP  (M:9, F:11)  44.2±10.6 years | 🗶 | ***NoLBP:*** subjects from outpatient hospital clinic & advertising,  *No LBP > 3months or at any time in previous year*  ***LBP:*** subjects from outpatient hospital clinic & advertising | ***Duration:*** > 6 months  ***Type:*** not stated  ***Pain/Activity limitation:***  VAS 29±25 | **yes** | *medium* | 3Space tracker (electro-magnetic)  ***Region:*** T1 and S1 | Proprioceptive position accuracy: flexion, extension & lateral flexion (reposition error ^o^) | Standing with pelvis restrained. Slow movement (5 seconds) flexion, extension & lateral flexion to 30, 60 & 90% of maximum ROM. One repeated measure of return to each position recorded |
| Ng,  2002 | 15NoLBP  15LBP  M  20-37years | ***NoLBP:***  22.7±2.0  ***LBP:***  23.4±1.9 | ***NoLBP:*** source not stated, *“Without any history of back pain”*  ***LBP:*** source not stated | ***Duration:*** >12 months duration  ***Type:*** not stated  ***Pain/Activity limitation***: severe enough to previously receive treatment, episodic or sustained pain. Minimal pain at time of testing  VAS 11±7%  RMDQ 10±8.3% | **Yes** | *low* | Inclinometer (Flexion, extension, lateral flexion  Rotameter (rotation)  ***Region:*** T12-S1 | Lordosis (^o^)  Flexion, extension, lateral flexion, rotation (^o^) | Warm-up procedure (1 repetition of each movement)  Pelvis restrained by device to eliminate pelvic/hip movement, 1 movement in each direction recorded |
| Norton  2004 | 60 NoLBP  128 LBP  19-73 years  (M:85, F:103) | 🗶 | ***NoLBP:*** friends, families of LBP subjects, local advertisement,  *No LBP previous 12 months*  ***LBP:*** from local physiotherapy clinics | ***Duration:*** not stated  ***Type:*** LBP +/- leg pain  ***Pain/Activity limitation:*** not stated | **Yes** | not stated | Metrocomm Skeletal Analysis System (3D)  ***Region:*** T12-S2 | Lordosis (^o^) | Maintain comfortable standing (lordosis)  Probe traced between points, repeated 3 times |
| Nourbakhsh2001 | 420 NoLBP  (M:210, F:210)  420 LBP  (M:210, F:210)  20-65years | ***NoLBP:***  M:*73.9±10.8kg*  F:*63.9±10.4*  ***LBP:***  M:*72.7±10.1*  F:*67.2±11.1* | ***NoLBP:*** *No LBP previous 12 months, no spinal surgery*  ***NoLBP & LBP:*** 8 metropolitan hospitals in Tehran | ***Duration:*** ≥ 6/52 low back pain (LBP) OR ≥ 3 episodes of LBP in previous year  ***Type:*** not stated  ***Pain/Activity limitation:*** not stated | **Mixed** | not stated | Flexible ruler  ***Region:*** T12-S2 | Lordosis (^o^) | Measured with flexible ruler using method of Youdas 1996 |
| O’Sullivan P  2003 | 15 NoLBP  (M:6, F:9)  38.2±10.9 years  15 LBP  (M:6, F:9)  38.8±12 years | ***NoLBP:***  *71.6±11.8 kg*  ***LBP:***  *73.9±18.4* | ***NoLBP:*** Recruited from local community, no LBP for*24 months*  ***LBP:*** recruited from private physiotherapy clinics | ***Duration:*** ≥ 3 months pain with  ***Type:*** “clinical lumbar segmental instability” flexion pattern  ***Pain/Activity limitation:***  ODI 26.1±13.3 % | **Yes** | *medium* | 3Space Fastrak (electro-magnetic)  ***Region:*** T12, L2, L4, S2 sensors | Repositioning error (absolute error) of the lumbar spine (^o^) | Sitting, with 3 repetitions of flexion to extension, then positioned in neutral position for 5 seconds. Subjects then relaxed into full flexion for 5 seconds then return to previous neutral position, x 5 |
| O’sullivan K  2013 | 15 NoLBP  (M:10, F:5)  32.1±9.2 years  15 LBP  (M:10, F:5)  31.3±10.3 years | ***NoLBP:***  23.8±2.0  ***LBP:***  24.3±3.2 | ***NoLBP:*** Recruited from local community, no LBP for*24 months*  ***LBP:*** recruited from private physiotherapy clinics | ***Duration:*** ≥ 3 months pain with  ***Type:*** “clinical lumbar segmental instability” flexion pattern  ***Pain/Activity limitation:***  VAS 33±19%  ODI 14.1±7.8 % | **Yes** | *medium* | Wireless posture monitor, strain gauge (BodyGuard)  ***Region:*** L3-S2 | Repositioning error (absolute error) of the lumbar spine (^o^)  Constant error and variable error | Sitting, then established full posterior tilt (flexion) to full anterior tilt (extension), then positioned in neutral position for 5 seconds. Subjects then relaxed into full flexion for 5 seconds then return to previous neutral position. 1 practice then 3 recorded trials |
| Paquet  1994 | 10 NoLBP  34±10 years  10 LBP  38±14 years | ***NoLBP:***  *79±12 kg*  ***LBP:***  *81±14* | ***NoLBP:*** Laboratory workers, *No detail provided*  ***LBP:*** Medical clinic outpatients | ***Duration:*** 7days to 7 weeks, ***Type:*** no leg pain  ***Pain/Activity limitation:***  VAS 32±13 | **Yes** | *medium* | Electro-goniometer (self-developed)  ***Region:*** T8-S1 | Flexion of lumbar spine & hip  (^o^) & (velocity: m/sec) | Comfortable standing, 5 flexion & return movements recorded at self-selected velocity, 5 flexion & return at specific velocity |
| Pope,  1985 (and Frymoyer, 1983) | 106 NoLBP  225 LBP  (144 moderate LBP, 71 severe LBP)  M  18-55 years | ***NoLBP:***  *78±11.3 kg*  Moderate ***LBP:***  *79.8±12.4*  Severe LBP  *81.1±13.7* | ***NoLBP & LBP:*** subjects sourced from large medical practice  *No detail provided* | ***Duration:*** not stated  ***Type:*** not stated  ***Pain/Activity limitation:*** categorised as moderate or severe (no other detail provided) | **Yes** | *“moderate” and “severe”* | Potentiometer  ***Region:*** T9 – S1 | Flexion, extension, lateral flexion, rotation (^o^) | Harness positioned at T9, attached to potentiometer, fixed pelvis . No other detail provided |
| Porter,  1997 | 17 NoLBP  15 LBP  M  18-36 years | 🗶 | ***NoLBP & LBP:***  No detail provided | ***Duration:*** Chronic LBP defined as episode of LBP. 49 days in previous 12months + current pain, +/- leg pain  ***Type:*** not stated  ***Pain/Activity limitation:*** categorised as moderate or severe (no other detail provided) | **Yes** | not stated | 3Space tracker (Polhemus)  ***Region:*** T12-S2 | Flexion, lumbar spine & hip (^o^) (hip versus lumbar contribution to trunk flexion, at 15^o^, 30^o^, 60^o^, 90^o^, & 120^o)^ | Flexion with extended knees from relaxed standing and return to standing, 2 practice movements, 1 recorded movement |
| Sheeran  2012 | 35 NoLBP  (M:13, F:22)  36±10.3 y  90 LBP  (M:21, F:59)  34.5±10.8 years | ***NoLBP:***  23.3±2.2  ***LBP:***  25.1±3.3 | ***NoLBP:***  no detail provided  ***LBP:*** sourced from people referred for physiotherapy to hospital board  Divided into flexion pattern (n=51) and active extension group (n=39) | ***Duration:*** >3 months,  ***Type:*** pain in lumbar or buttock region, clear mechanical basis with aggravating & easing movement directions, pattern of flexion or extension  ***Pain/Activity limitation***: Flexion group: RMDQ 30±16 %, current pain 48±13%  (Active) Extension group:  RMDQ 26±15 %, current pain 45±14% | **Yes** | *medium* | Opto-electronic (Vicon) and computer-assisted, mechanical (Spinal Mouse)  ***Region:*** L1-L5 *(and T1-T12)* | Flexion, extension re-positioning error  Absolute error (magnitude), variable error (consistency) & constant error (direction) (^o^) | Subjects seated, blindfolded, performed 3 flexion & extension movements, then placed in a mid-range, neutral position for 5 seconds to be memorised. Subjects relaxed in usual sitting 5 seconds then attempted to reproduce memorised position 4 times. Process repeated in standing. |
| Sung,  2012 | 15NoLBP  41.8±16.88  15LBP  47.9±13.8 years  (M:14&F:16) | ***NoLBP:*** *70.5±8.* *kg*  ***LBP:*** *64.8±10.5* | ***NoLBP & LBP:***  No detail provided | ***Duration:*** > 2 months  ***Type:*** no leg pain  ***Pain/Activity limitation:***  ODI 20±48% (range 0-37%) | **Yes** | not stated | Motion analysis labortatory, reflective markers  ***Region:*** T12-S2 (and thoracic spine measured separately) | Axial rotation of upper thorax, lower thorax and lumbar regions (^o^) | Standing upright, holding bar at shoulder height then rotating body, knees extended, feet fixed, 5 repetitions |
| Taimela,  1999 | 49 NoLBP  (M:28, F:21)  38±9 y  57 LBP  (M:27, F:30)  41±7 years | ***NoLBP:*** M:*80.9±9.2 kg,*  *F:64±7.2*  ***LBP:***  M:*82.6±14*  *F:64.5±8.9* | ***NoLBP & LBP:*** sourced via advertisement in local newspapers  *No significant LBP requiring medical attention in previous 2 years* | ***Duration:*** >3 months  ***Type:*** non-specific LBP  ***Pain/Activity limitation:*** Male:  VAS 52±19%,  ODI 21±9%,  Female:  VAS 61±23%, ODI 26±16% | **Yes** | *high* | Specifically manufactured rotating seat  ***Region:*** Lumbar spine ( non- specific) | Propioception of axial rotation in lumbar spine (^o^)  Motion perception threshold (msec) | Subjects blindfolded, seated in neutral position, holding a switch, which is pressed when movement (rotation) detected in lumbar spine. Seat rotates at 1^o^/sec. Standardized practice then measurement of magnitude of seat rotation recorded on 5 repetitions. Two tests, before and after fatiguing process with resisted flexion/extension exercise |
| Tsai  2010 | 16NoLBP  47.9±8.3  16LBP  48.6±7.4 years  (M:14&F:16) | ***NoLBP:*** *87.5±9.6* *kg*  ***LBP:*** *88.3±18.2* | ***NoLBP:*** No detail provided  ***LBP:*** | ***Duration:*** one episode within last 2 years with ODI>24% & required conservative treatment  ***Type:*** “mechanical LBP”  ***Pain/Activity limitation:***  VAS 0  ODI 45.3±18.2% (at time of episode) | **No** | *NA* | Opto-electronic (Vicon)  ***Region:*** T1 – S1 | Flexion, extension, lateral flexion , rotation (^o^),  Flexion, extensión, lateral flexion & rotation (left & right) re-positioning error (absolute) (^o^) | Subjects stood with pelvis immobilised, one repetition of full ROM in each direction (recorded for ROM comparison), blindfolded, moved to 8-% maximum ROM for 4 seconds, returned to neutral then asked to move back to target x6 for each direction |
| Waddell, 1992 | 70NoLBP  120LBP  M&F  20-55 | 🗶 | ***NoLBP:*** from hospital patients with hand injuries, hospital visitors and staff, *no current pain nor history of LBP requiring medical attention of time off work in previous months*  ***LBP:*** subjects from orthopaedic outpatient clinic | ***Duration:*** > 3 months  ***Type***: +/- thigh pain but no radiculopathy signs  ***Pain/Activity limitation:*** collected but not reported | **Yes** | not stated | Electric inclinometer (Cybex / Lumex)  ***Region:*** T12-S1 | Flexion, extension, lateral flexion (^o^) | Flexion, Extension, Rotation, Lateral flexion x2 as warm-up. Third repetition recorded. Standardised position and instruction. |
| Willigenburg  2012 | 13 NoLBP  (M:9, F:4)  34.3±11.9 y  20 LBP  (M:11, F:9)  33.4±15.5 years | ***NoLBP:***  22.9±2.4  ***LBP:***  23.6±3 | ***NoLBP & LBP:***  No detail provided | ***Duration:*** > 6 weeks  ***Type***: non specific LBP  ***Pain/Activity limitation:***  VAS 27±19% | **Yes** | *medium* | Opto-electronic  (OptoTrak)  ***Region:*** T12 and pelvic marker | Deviation from neutral position   - % time on target - Accuracy (average ^o^ change from initial angle) - Precision (used SDs) | Kneel sitting position, adopting a “neutral” posture while watching realtime visual biofeedback with a black dot representing actual position. Subjects had to keep the dot contained within a small square (0.2o range) representing high precision trunk control then then large square (2.7o range) for 30 seconds representing low precision control |
| Willigenburg  2013 | 13 NoLBP  (M:9, F:4)  34.3±11.9 y  18 LBP  (M:11, F:9)  31±14 years | ***NoLBP:***  22.9±2.4  ***LBP:***  23.4±2.4 | ***NoLBP & LBP:***  No detail provided | ***Duration:*** > 6 weeks  ***Type***: non specific LBP  ***Pain/Activity limitation:***  ODI 15.2±4.2  VAS 27±19% | **Yes** | *medium* | Opto-electronic  (OptoTrak)  ***Region:*** T12 and pelvic marker | Tracking error (absolute difference between trunk angle and target angle) | Kneel sitting position, adopting a “neutral” posture while watching realtime visual biofeedback with a black dot representing actual position. Subjects were asked to keep black dot located within a yellow rectangle which was programmed to move in a spiral trajectory of 5 circles. Task took 2 minutes. 2 trials starting at centre of spiral moving outwards and 2 at end of spiral moving inwards. Angles in sagittal & frontal planes calculated |
| Wong,  2004 | 20 NoLBP  42±8 y  24LBP (group 2)  41±11 years  21 LBP+ive SLR (group 3)  34±10 years | ***NoLBP:*** *71.4±10.5 kg*  ***LBP:*** Group 2 *68.6±5.5*  Group 3: *71.4±4.5.* | ***NoLBP & LBP:*** sourced from university & outpatient physiotherapy clinic  *No significant LBP or leg pain in previous year* | ***Duration:*** not stated  ***Type***: back pain only or back + positive straight leg raise  ***Pain/Activity limitation:***  Group 2  RMDQ: 42±16, VAS 60±20%,  Group 3  RMDQ: 50±16, VAS 60±20% | **Yes** | *high* | 3Space Fastrak (electro-magnetic)  ***Region:*** L1 and S2, bilateral hips | Flexion, extension (Lx & hip), lateral flexion and rotation (Lx only) (^o^), velocity (deg/sec) | Warm-up flexion, extension, lateral flexion & rotation in comfortable standing. 3 repetitions recorded at self-selected pace |
| Youdas  1996, 2000 | 90 NoLBP  (M:45, F:45)  40-70 years  60 LBP  (M:30, F:30)  40-70 years | ***NoLBP:*** M:26.6±3.5  F: 26.1±5  ***LBP:***  M:26.9±3.6  F:28.9±5.7 | ***NoLBP:*** personal contact, personnel from Mayo clinic, ad in newspaper  *No current LBP, no surgery, no history of hospitalisation for LBP*  ***LBP:*** subjects sourced from local advertisement at institute, newspaper | ***Duration:*** > 4 months  ***Type:*** not stated  ***Pain/Activity limitation:***  ODI M: 15±9.5, F:26.7±9.7 | **Yes** | not stated | Inclinometer (pelvic tilt)  Flexible rule for all other measurements  ***Region:*** T12-S2 | Pelvic inclination (angle from vertical), lumbar lordosis, flexion, extension (^o^) | Comfortable standing (pelvic tilt, lordosis)  Sitting, instructed to “place head between knees” x 3 as preparation then 1 recorded repetition (flexion)  Prone, press up with hips on couch 1 repetition (extension) |

NOLBP no low back pain; LBP low back pain; NSLBP non-specific low back pain; F flexion; E extension; LF lateral flexion; rot rotation; VAS 100 point visual analogue scale for pain intensity; RMDQ = Roland Morris Disability Questionnaire for activity limitation (converted to %); 🗶 not reported
