# Supplementary material for: Comparing lumbo-pelvic kinematics in people with and without back pain: a systematic review and meta-analysis
Source: BMC Musculoskelet Disord. 2014 Jul 10;15:229. doi: 10.1186/1471-2474-15-229 (PMC4096432; doi:10.1186/1471-2474-15-229)
Supplement: Additional file 5 — Quality assessment. [file 1471-2474-15-229-S5.docx]

# Additional file 5: Quality Assessment

## Quality assessment results

|  |  | **Selection bias** | | | | | | **Measurement and outcome bias** | | | | | | | |  |  |
| --- | --- | --- | --- | --- | --- | --- | --- | --- | --- | --- | --- | --- | --- | --- | --- | --- | --- |
|  | **Study (1^st^ author, date)** | Was the study population adequately described? | Where both groups drawn from the same population (or age/sex matched)? | Were both groups comparable for age, sex, BMI/weight | Was pain intensity and/or activity limitation described for LBP group? | Were characteristics of those with back pain described? | **Score for selection bias (Maximum score = 5)** | Description enables accurate replication of the measurement procedures | Instrument used to measure described | A system for standardising movement instructions is reported | Were assessors trained in standardised measurement procedure? | Did the same assessors test those with and without back pain? | Were assessors blinded as to which group subjects were in? | Was the same assessment procedure applied to those with and without back pain? | **Score for measurement and outcome bias (maximum score = 7)** | Between-group statistical comparisons are reported for at least one key outcome | **Total Score** |
|  | Aluko  2011 | Y | n | Y | Y | Y | **4** | Y | Y | n | n | n | n | Y | **3** | n | **7** |
|  | Barrett  1999 | n | n | Y | n | n | **1** | Y | Y | n | n | n | n | Y | **3** | Y | **4** |
|  | Boline  1992 | Y | Y | n | n | n | **2** | Y | Y | n | Y | Y | n | n | **4** | n | **6** |
|  | Brumagne  2000 | Y | n | Y | Y | n | **3** | Y | Y | n | n | n | n | Y | **3** | Y | **6** |
|  | Christie  1995 | n | n | n | n | Y | **1** | Y | Y | Y | n | n | n | Y | **4** | Y | **5** |
|  | Crosbie  2013 | Y | n | Y | Y | n | **3** | n | Y | n | n | n | n | Y | **2** | Y | **5** |
|  | Day  1984 | n | n | n | n | n | **0** | Y | Y | Y | n | Y | n | Y | **5** | Y | **5** |
|  | Descarreaux 2005 | Y | Y | Y | Y | n | **4** | n | n | n | n | n | n | Y | **1** | Y | **5** |
|  | Esola  1996 | Y | n | Y | Y | n | **3** | Y | Y | Y | n | n | n | Y | **4** | Y | **7** |
|  | Field  1997 | n | Y | n | n | n | **1** | Y | Y | n | n | Y | n | Y | **4** | Y | **5** |
|  | Georgy  2011 | n | Y | n | Y | n | **2** | Y | Y | n | n | n | n | Y | **5** | Y | **7** |
|  | Gill  1998 | n | n | n | n | n | **0** | Y | Y | Y | n | Y | n | Y | **5** | Y | **5** |
|  | Gomez  1994 | Y | n | n | n | n | **1** | n | Y | Y | n | n | n | Y | **4** | Y | **5** |
|  | Hidalgo  2012 | Y | n | Y | n | Y | **3** | Y | Y | n | n | n | n | Y | **3** | Y | **6** |
|  | Hidalgo  2013 | Y | n | Y | Y | n | **3** | Y | Y | n | n | n | n | Y | **3** | Y | **6** |
|  | Hultman  1993 | Y | Y | Y | n | n | **3** | Y | Y | Y | n | Y | n | Y | **5** | Y | **8** |
|  | Kim  2013 | n | n | n | n | Y | **1** | Y | Y | n | n | n | n | n | **2** | Y | **3** |
|  | Koumantakis  2002 | n | n | n | Y | n | **1** | Y | Y | n | n | n | n | n | **2** | Y | **3** |
|  | Lee  2010 | Y | n | Y | Y | n | **3** | Y | Y | Y | n | n | n | Y | **4** | Y | **7** |
|  | Marras  1995 | n | n | n | n | Y | **1** | Y | Y | Y | n | n | n | Y | **4** | Y | **5** |
|  | McClure  1997 | Y | n | Y | Y | n | **3** | Y | Y | Y | n | n | n | Y | **4** | Y | **7** |
|  | McGregor 1995,1997 | n | n | n | Y | Y | **2** | Y | Y | n | n | n | n | Y | **3** | Y | **5** |
|  | McGregor 2000 | n | Y | n | n | Y | **2** | Y | Y | n | n | n | n | Y | **3** | Y | **5** |
|  | Mellin  1990 | n | Y | n | n | n | **1** | Y | Y | n | n | n | n | Y | **3** | Y | **4** |
|  | Newcomer 2000A | n | Y | n | Y | n | **2** | Y | Y | n | n | Y | n | Y | **4** | Y | **6** |
|  | Newcomer  2000B | n | Y | n | Y | n | **2** | Y | Y | n | n | Y | n | Y | **4** | Y | **6** |
|  | Ng  2002 | Y | n | Y | Y | n | **3** | Y | Y | Y | n | n | n | Y | **4** | Y | **7** |
|  | Norton  2004 | n | Y | n | n | n | **1** | Y | Y | n | Y | n | n | Y | **4** | Y | **5** |
|  | Nourbakhsh 2001 | Y | Y | Y | n | n | **3** | Y | Y | Y | n | n | n | Y | **4** | Y | **7** |
|  | O’Sullivan P  2003 | Y | Y | Y | Y | Y | **5** | Y | Y | n | n | n | n | Y | **3** | Y | **8** |
|  | O’Sullivan K  2013 | Y | Y | Y | Y | Y | **5** | Y | Y | n | n | n | n | Y | **3** | Y | **8** |
|  | Paquet  1994 | n | n | Y | Y | Y | **3** | Y | Y | n | n | n | n | Y | **3** | Y | **6** |
|  | Pope  1985 | Y | n | n | n | n | **1** | Y | Y | Y | n | n | n | Y | **4** | n | **5** |
|  | Porter  1997 | n | n | n | n | Y | **1** | Y | Y | Y | n | n | n | n | **3** | Y | **4** |
|  | Sheeran  2012 | Y | n | n | Y | Y | **3** | Y | Y | n | n | Y | n | Y | **4** | Y | **7** |
|  | Sung  2012 | Y | Y | Y | n | Y | **4** | n | n | n | n | n | n | Y | **1** | Y | **5** |
|  | Taimela  1999 | Y | Y | Y | Y | n | **4** | Y | Y | Y | n | n | n | Y | **4** | Y | **8** |
|  | Tsai  2010 | n | Y | Y | Y | n | **3** | Y | Y | n | n | n | n | Y | **3** | Y | **6** |
|  | Waddell  1992 | n | Y | n | n | Y | **2** | Y | Y | Y | n | n | n | Y | **4** | Y | **6** |
|  | Willigenburg 2012 | n | n | Y | Y | n | **2** | Y | Y | n | n | n | n | Y | **3** | Y | **5** |
|  | Willigenburg 2013 | Y | n | Y | Y | n | **3** | Y | Y | n | n | n | n | Y | **3** | Y | **6** |
|  | Wong  2004 | n | Y | Y | Y | n | **3** | Y | Y | n | n | n | n | Y | **3** | Y | **6** |
|  | Youdas 1996, 2000 | Y | n | Y | Y | n | **3** | Y | Y | n | n | n | n | Y | **3** | Y | **6** |
| **TOTAL Score &**  **Percentage Yes %** | | **23/41 (57%)** | **16/41**  **(39%)** | **22/41**  **(54%)** | **23/41**  **(56%)** | **14/41**  **(34%)** | **2.4/5**  **48% avg.** | **37/41**  **(90%)** | **39/41**  **(95%)** | **15/41**  **(37%)** | **2/41**  **(2%)** | **7/41**  **(17%)** | **0**  **(0%)** | **38/41**  **(93%)** | **3.4/7**  **49% avg.** | **38**  **(94%)** | **5.8/12**  **48% avg.** |
