# Supplementary material for: Comparing lumbo-pelvic kinematics in people with and without back pain: a systematic review and meta-analysis
Source: BMC Musculoskelet Disord. 2014 Jul 10;15:229. doi: 10.1186/1471-2474-15-229 (PMC4096432; doi:10.1186/1471-2474-15-229)
Supplement: Additional file 6 — Summary of studies examining lumbar proprioception. [file 1471-2474-15-229-S6.docx]

Additional file 6: Summary of studies examining lumbar proprioception

|  | **Author, Date** | **Position** | **Type of test** | **Region measured** | **Movement direction tested** | **Movement kinematics measured** | **Angle of re-positioning** | **Number of test movts before data collection** | **Comments**  **(to be deleted)** |
| --- | --- | --- | --- | --- | --- | --- | --- | --- | --- |
|  | Brumagne  2000 | sitting | re-position | S2 | Sagittal | Pelvic tilt re-position error in sitting (constant, variable and absolute error) | Neutral sitting | 1 |  |
|  | Descarreaux 2005 | standing | re-position | “trunk” | Sagittal | Standing flexion (15^o^, 30^o^, 60^o^), extension (15^o^) reposition accuracy  Temporal symmetry | 15,30, 60^o^ flexion,  15^o^ extension | Yes, unlimited until accuracy (within 10%) was achieved | No means /SDs on absolute error or differences between groups  Stated no diff between groups (but after diff number of practice trials  Which area was tested? |
|  | Georgy, 2011 | sitting | re-position | T1-S2 | Sagittal | Repositioning error (absolute error) of the thoracolumbar spine (^o^) | 30^o^ flexion | 3 | Instrument used did not differentiate between Lx and Tx |
|  | Gill  1998 | standing,  4 point kneeling | re-position | T12-S1 | Sagittal | Proprioceptive position accuracy of position/reposition at 20^o^ flexion in standing & 4-point kneeling |  | 10 |  |
|  | Hidalgo, 2013 | sitting | re-position | T12-S2 | Sagittal | Repositioning error (absolute error) of the lumbar spine (^o^) | 30 ^o^ flexion | 1 |  |
|  | Koumantakis  2002 | Standing | re-position | T12-S2 | Sagittal  Transverse  Frontal | Repositioning error (absolute error) of the lumbar spine (^o^), | flexion 20^o^, rotation , lateral flexion to 15^o^ | unknown |  |
|  | Lee  2010 | Sitting, sidelying, supine | re-position  Motion perception threshold | Thoraco-lumbar (not clearly stated) | Sagittal  Transverse  Frontal | Repositioning error (absolute error) of the lumbar spine (^o^)  Motion perception threshold (^o^) | 15 ^o^ flexion | 2 | Not clear about area tested  Unusual position |
|  | Newcomer, 2000A | standing | re-position | L1 and S1 | Sagittal  Transverse  Frontal | Proprioceptive position accuracy:  flexion, extension, lateral flexion & rotation in standing (reposition error ^o^) | 50% max ROM | 1 |  |
|  | Newcomer, 2000B | standing | re-position | T1 and S1 | Sagittal  Transverse  Frontal | Proprioceptive position accuracy: flexion, extension & lateral flexion (reposition error ^o^) | 30, 60, 90% max ROM | 1 | Full trunk not just Lx |
|  | O’Sullivan,P 2003 | sitting | re-position | T12, L2, L4, S2 sensors | Sagittal | Repositioning error (absolute error) of the lumbar spine (^o^) | Neutral sitting | 1 | Flexion control impairment group (sub group of LBP subjects) |
|  | O’Sullivan,K 2013 | sitting | re-position | L3-S2 | Sagittal | Repositioning error (absolute error) of the lumbar spine (^o^)  Constant error and variable error | Neutral sitting | 1 | Flexion control impairment group (sub group of LBP subjects) |
|  | Sheeran, 2012 | standing, sitting | re-position | L1-L5 and T1-T12 | Sagittal | Flexion, extension re-positioning error  Absolute error (magnitude), variable error (consistency) & constant error (direction) (^o^) | Neutral | 1 | Flexion control impairment and active extension impairment. No differences between sub-groups for absolute error but constant error was different.  sEMG shows higher muscle activation for pain group.  No diff between AE & F groups  Thoracic spine showed sig difference between normal and pain groups but of a lower magnitude |
|  | Taimela, 1999 | sitting | Motion perception threshold | Lumbar spine ( non- specific) | Transverse | Propioception of axial rotation in lumbar spine (^o^)  Motion perception threshold (msec) | NA | Not stated | Fatigue makes movt detection less sensitive |
|  | Willigenburg 2012 | Kneel-sitting | Motion control  precision | T12 and pelvic marker |  | Deviation from neutral position   - % time on target - Accuracy (average ^o^ change from initial angle) - Precision (used SDs) | NA | 1 | Discarded first 5 seconds of the trial |
|  | Willigenburg2013 | Kneel-sitting | Motion tracking precision | T12 and pelvic marker |  | Tracking error (absolute difference between trunk angle and target angle) | NA | 1 | Discarded first 5 seconds of the trial |
